# Effective Targeting of Colon Cancer Cells with Piperine Natural Anticancer Prodrug Using Functionalized Clusters of Hydroxyapatite Nanoparticles

**DOI:** 10.3390/pharmaceutics12010070

**Published:** 2020-01-16

**Authors:** Khaled AbouAitah, Agata Stefanek, Iman M. Higazy, Magdalena Janczewska, Anna Swiderska-Sroda, Agnieszka Chodara, Jacek Wojnarowicz, Urszula Szałaj, Samar A. Shahein, Ahmed M. Aboul-Enein, Faten Abou-Elella, Stanislaw Gierlotka, Tomasz Ciach, Witold Lojkowski

**Affiliations:** 1 Laboratory of Nanostructures, Institute of High Pressure Physics, Polish Academy of Sciences, Sokolowska 29/37, 01-142 Warsaw, Poland; a.swiderska-sroda@labnano.pl (A.S.-S.); a.chodara@labnano.pl (A.C.); j.wojnarowicz@labnano.pl (J.W.); u.szalaj@labnano.pl (U.S.); xray@unipress.waw.pl (S.G.); w.lojkowski@labnano.pl (W.L.); 2 Medicinal and Aromatic Plants Research Department, Pharmaceutical and Drug Industries Research Division, National Research Centre (NRC), P.C. 12622 Dokki, Giza, Egypt; 3 Biomedical Engineering Laboratory, Faculty of Chemical and Process Engineering, Warsaw University of Technology, 00-645 Warsaw, Poland; Agata.Stefanek.dokt@pw.edu.pl (A.S.); Magdalena.Janczewska.dokt@pw.edu.pl (M.J.); Tomasz.Ciach@pw.edu.pl (T.C.); 4 Department of Pharmaceutical Technology, Pharmaceutical and Drug Industries Research Division, National Research Centre (NRC), P.C. 12622 Dokki Giza, Egypt; imane.higazy@hotmail.com; 5 Faculty of Materials Engineering, Warsaw University of Technology, Wołoska 41, 02-507 Warsaw, Poland; 6 Biochemistry Department, Faculty of Agriculture, Cairo University, P.C. 12613 Giza, Egyptaboul.enein1@gmail.com (A.M.A.-E.); faten2010bio@yahoo.com (F.A.-E.)

**Keywords:** hydroxyapatite nanocarrier, piperine alkaloid prodrug, natural products, delivery system for cancer targeting, nanoformulations, colon cancer cells and spheroids, in vitro release kinetics, pH-sustained release effect, folic acid

## Abstract

Targeted drug delivery offers great opportunities for treating cancer. Here, we developed a novel anticancer targeted delivery system for piperine (Pip), an alkaloid prodrug derived from black pepper that exhibits anticancer effects. The tailored delivery system comprises aggregated hydroxyapatite nanoparticles (HAPs) functionalized with phosphonate groups (HAP-Ps). Pip was loaded into HAPs and HAP-Ps at pH 7.2 and 9.3 to obtain nanoformulations. The nanoformulations were characterized using several techniques and the release kinetics and anticancer effects investigated in vitro. The Pip loading capacity was >20%. Prolonged release was observed with kinetics dependent on pH, surface modification, and coating. The nanoformulations fully inhibited monolayer HCT116 colon cancer cells compared to Caco2 colon cancer and MCF7 breast cancer cells after 72 h, whereas free Pip had a weaker effect. The nanoformulations inhibited ~60% in HCT116 spheroids compared to free Pip. The Pip-loaded nanoparticles were also coated with gum Arabic and functionalized with folic acid as a targeting ligand. These functionalized nanoformulations had the lowest cytotoxicity towards normal WI-38 fibroblast cells. These preliminary findings suggest that the targeted delivery system comprising HAP aggregates loaded with Pip, coated with gum Arabic, and functionalized with folic acid are a potentially efficient agent against colon cancer.

## 1. Introduction

In 2018, 1.9 million cancer-related deaths and 3.9 million new cases were reported in Europe, with an increase in cancer incidence and death rates expected [1]. The main treatments for cancer include chemotherapy, radiotherapy, and surgery. However, chemotherapy results in toxicity towards normal cells and the development of therapy-resistant cancers. Integrating the above methods is not sufficiently effective [2,3]. Therefore, active targeting of cancer cells with anticancer drugs has been explored.

Natural medicines account for 60% of anticancer agents used clinically [4]. Natural products isolated from plants (also called natural prodrugs)—such as vincristine, taxanes, and camptothecin—have a long history in the treatment and prevention of cancer. Natural prodrugs offer safe, cost-effective, and diverse biological-medicinal activities [5,6]. Despite these advantages, only a few natural prodrugs are in clinical use because of several limitations, specifically poor water solubility, low bioavailability, short half-life, and non-specific targeting. Nanomedicine has the potential to offer solutions to circumvent these limitations [7,8,9].

Among anticancer natural plant-derived prodrugs is piperine (Pip), an alkaloid of special interest. Pip is an amide alkaloid extracted and isolated from the fruits of the black pepper plant (*Piper nigrum* Linn). Black pepper has a top position among other spices and kitchen uses due to its unique pungency and flavor, earning it the nickname “the king of spices” [10,11]. The average of consumption of black pepper is equivalent to 16 mg to 30 mg of pure Pip per person per day [12]. Black pepper contains 6% to 9% pure Pip based on the dry weight [11]. Pip has a long history of use in traditional Chinese, Indian, and Arabic medicine as a remedy for many illnesses (e.g., pain alleviation, indigestion, chills, rheumatism, infection, influenza, fever). At the pharmacological level, Pip improves the bioavailability of drugs, either synthetic or natural, when combined [13,14], and has anti-inflammatory [15], neuroprotective [16], and anti-oxidant effects [17]. Anti-tumor effects have been reported in several cancers both in vitro and in vivo, including melanoma, breast, ovarian, colon, lung, liver, and prostate [18,19,20,21,22,23,24]. Despite these fascinating properties, Pip has not yet been used clinically due to its inherent limitations in regards to solubility, site-specific targeting, and bioavailability.

**Scheme 1:**
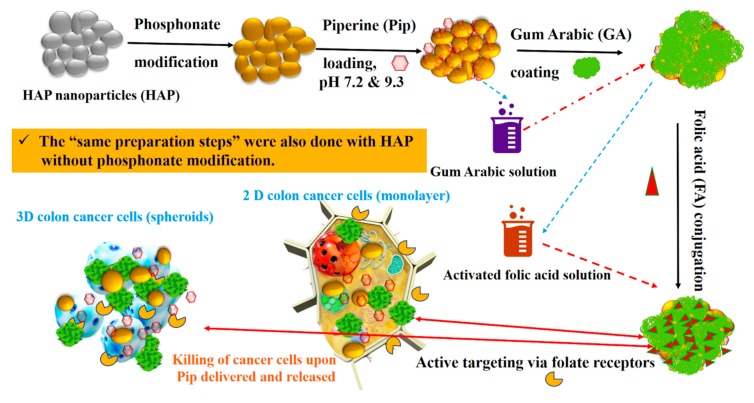
Schematic representation of preparation steps for the proposed delivery system using the natural prodrug of piperine and HAPs to target colon cancer cells in monolayer and spheroids.

Recently, many strategies have been developed for Pip delivery systems using different nanoparticles, including chitosan-sodium tripolyphosphate [25], solid lipids [26], PLGA [27], chitosan nanoparticles [28], and self-emulsifying drug delivery [29]. However, most of the delivery systems developed for Pip have investigated polymeric or lipid nanocarriers. The above-mentioned systems have limitations as far as long-term release effect is considered. Release of over 90% of encapsulated Pip takes place within few hours. This effect is expected since release depends on degradation of the polymeric drug carrier such as chitosan, which takes place in short time. Inorganic nanocarriers used in drug delivery systems (DDSs) for drugs and therapeutic agents have included mesoporous silica nanoparticles (MSNs), magnetic nanoparticles, zinc nanoparticles, and others to treat various diseases [30,31,32,33]. Hydroxyapatite is the primary inorganic component of teeth and bone. They exhibit a good biocompatibility compared to other inorganic materials that biodegrade in biological fluids. With such fascinating properties, hydroxyapatite nanoparticles have shown many efforts in the delivery of drug and therapeutic agents with prolonged release [34,35,36,37,38,39]. Hydroxyapatite nanoparticles (HAPs) are of great interest in biomedical applications, such as bone regeneration [40,41]. Though HAPs have many applications as vehicles, especially in anticancer therapy, they have not yet designed DDSs for important prodrugs for cancers through active targeting. In the current study, we established a novel DDS comprising HAPs as nanocarriers loaded with Pip, followed by coating with gum Arabic (GA) and conjugation of folic acid (FA) on the surface as active targeting ligands for cancer selectivity (Scheme 1). The delivery system shows promising and potential anticancer effects against monolayer (two dimensions) and spheroid (three dimensions) HCT116 colon cancer cells. The current study demonstrates a prolonged release effect (over 90 h) for Pip from loaded into agglomerated HAP nanoparticles. It depends on pH conditions and total Pip content and is considerably longer than for organic delivery systems for delivering Pip. Such a long-term release effect is required for cancer therapy. Furthermore, our system shows quite a high drug loading capacity of over 20 wt%, which may be superior over the existing systems. Therefore, the current results show a possible way to deliver Pip to target colon cancer cells with a prolonged release effect.

## 2. Materials and Methods

### 2.1. Synthesis and Surface Modification of Hydroxyapatite Nanoparticles

HAPs were synthesized using the hydrothermal method according to Kuśnieruk et al. [42]. The surface of the HAP was functionalized with phosphonate (P) groups by adding 1.5 g of HAPs to 100 mL of 18.2 MΩ ultra-pure water (Milli-Q^®^ system, Millipore, Darmstadt, Germany) containing 1.5 mL of 3-(trihydroxysilyl)propyl methylphosphonate monosodium salt solution (Santa Cruz Biotechnology, Dallas, TX, USA) with stirring at room temperature. This solution was left for 24 h, and then filtered, washed several times with deionized water, and dried at 60 °C for 24 h. In this condition, the post functionalization of HAP with P groups is possible via the interaction hydrogen bonding of P to a hydroxyl group.

### 2.2. Piperine Loading

The Pip loading ratio was 1:3 (drug: nanoparticle); 300 mg Pip (Sigma-Aldrich, St. Louis, MO, USA) was dissolved in 20:80 ultrapure water:ethanol (Fisher Scientific, Loughborough, UK) at room temperature, followed by 900 mg of HAPs and HAP-Ps. The pH adjusted to either 7.2 or 9.3. The mixture solution was stirred for 24 h, and then evaporated at 60 °C (Rotavap; Büchi, Flawil, Switzerland). The dried mixture was resuspended in ultrapure water several times to remove unloaded molecules before drying in a 60 °C oven for 12 h. The obtained product was named HAP-Pip7.2, HAP-Pip9.3, HAP-P-Pip7.2, and HAP-P-Pip9.3. These materials were intended for coating with GA in subsequent steps.

### 2.3. Gum Arabic Coating and Folic acid Conjugation

For GA coating, GA solution (1%) was prepared by dissolving GA powder (Acros Organics, Geel, Belgium) in 0.1 M of NaOH (Acros Organics, Geel, Belgium) under high speed stirring at 60 °C. Next, 400 mg of HAP-Pip9.3 or HAP-P-Pip9.3 was suspended in GA solution and stirred at 250 rpm for 72 h at room temperature. The solution was centrifuged, washed several times with deionized water until the pH of the solution was neutral (7 to 7.4), and then dried at 60 °C in an oven for 12 h. The resulting products were named HAP-Pip9.3-GA and HAP-P-Pip9.3-GA.

For FA conjugation, we prepared the GA coating using the same procedure but without the drying step. Subsequently, the GA-coated HAPs (not dry) were suspended in the activated FA solution. The FA-activated solution was prepared in a separate step by adding 100 mg of FA (Sigma-Aldrich, St. Louis, MO, USA) and 50 mg of EDC (Acros Organics, Geel, Belgium) in 20 mL of NaOH (0.1 M) with stirring (400 rpm) at 60 °C. Typically, GA-coated particles were suspended in 10 mL of FA solution and stirred at 275 rpm for 40 h at room temperature. The solution was centrifuged to collect the particles, which were then washed several times to remove unbonded FA and EDC molecules. The obtained product was dried at 60 °C in an oven for 12 h and named HAP-Pip9.3-GA-FA and HAP-P-Pip9.3-GA-FA. The materials were stored at room temperature until further use.

Investigations of total drug-loading capacity (TLC) and entrapment efficiency (EE) using UV–vis and thermogravimetric (TG) analysis are detailed in the Supplementary Information.

### 2.4. Characterization Techniques

The following techniques were used to characterize the resulting nanomaterials: field emission scanning electron microscopy (FE-SEM; Ultra Plus, Zeiss, Jena, Germany); powder X-ray diffraction (XRD; X’PertPRO System, PANalytical, Marietta, GA, USA) utilizing CuKα radiation (2θ range of 10–100°); Fourier transform infrared (FTIR) spectroscopy (Bruker Optics Tensor 27, Bruker Corporation, Billerica, MA, USA) equipped with attenuated total reflectance (Platinium ATR-Einheit A 255); simultaneous thermal analysis (STA) coupled with differential scanning calorimetry (DSC; STA 499 F1Jupiter, NETZSCH-Feinmahltechnik GmbH, Selb, Germany); the NOVA automated gas sorption system from Quanta Chrome Instruments (Boynton Beach, FL, USA) to measure pore size distribution; and the Brunauer, Emmett, and Teller (BET) specific surface area analysis (Gemini 2360, Micromeritics, Norcross, GA, USA) according to ISO 9277:2010. The powders were dried at 150 °C (without drug) or 50 °C (with drug and polymer) for 24 h under constant helium flow (FlowPrep 060 desorption station, Micromeritics) before analysis for additional confirmation. In STA-DSC, samples (5–10 mg) were placed in the STA unit’s alumina pan. Before measurement, helium flowed for 30 min through the STA furnace chamber. The heating rate was 10 °C/min in a helium/air mixture to 850 °C. Zeta potential was determined using a NanoZS Malvern ZetaSizer (Malvern, UK) based on a phosphate-buffered saline (PBS) suspension of nanoparticles (adjusted for different pH) at 24 °C.

The detailed procedures for measuring solubility are provided in the Supplementary Information.

### 2.5. In Vitro Drug Release

We studied the dissolution profile of Pip using a bottle method with a cellulose dialysis bag (MWCO 12,000 g/mol, Sigma-Aldrich CHEMIE GmbH, Sternheim, Germany). Each bag contained 3 mL of PBS with Pip-loaded HAPs and was closed from both sides before immersion in a capped glass bottle containing 50 mL of PBS adjusted for different pH: 5, 6.8, or 7.4. Bottles were incubated at 37 °C ± 0.5 °C in a WNB14 Memmert Shaking Water Bath (INDO Gama Pratama, Yogyakarta, Indonesia) with the rotation speed fixed at 150 rpm (Erweka GmbH, Hessen, Germany). We added 0.01% *w*/*v* sodium azide to the release media as a preservative. At 6, 8, 12, 24, 48, and 72 h, a 2 mL aliquot was withdrawn, filtered through a 0.45-mm Millipore filter, and measured at 342 ± 0.05 nm against the blank cuvette. We maintained sink conditions by adding an equal volume of fresh buffer with each sampling. Each experiment was carried out in triplicate. The drug release data were analyzed using KinetDS3 pharmacokinetics software from Jagiellonian University (Department of Pharmaceutical Technology and Biopharmaceutics, Faculty of Pharmacy), employing both linear and non-linear regression. Release efficiency (RE) and mean dissolution time (MDT) were also determined [43].

### 2.6. Cell Culture

We used MCF 7 human breast adenocarcinoma cells and Caco2 human colon carcinoma cells, with WI-38 human fibroblast cells as the normal reference (American Type Culture Collection [ATCC], Manassas, VA, USA). The cells were maintained and tested at the Confirmatory Diagnostic Unit (VACSERA, Dokki, Giza, Egypt). These three cell lines were cultured in Dulbecco’s modified Eagle medium (DMEM) supplemented with 10% fetal bovine serum, streptomycin (100 μg/mL), and penicillin G (100 U/mL; all from Gibco, Thermo Fisher Scientific) at 37 °C in a humidified atmosphere containing 5% CO_2_. For in vitro biological evaluations, we used HCT116 human colon cancer cells (Sigma-Aldrich, ECACC, USA) due to the high FA receptor content at the cell surface. The HTC116 cells were cultured in McCoy’s 5a medium supplemented with 10% fetal bovine serum, streptomycin (100 μg/mL), and penicillin G (100 U/mL) at 37 °C in a humidified atmosphere containing 5% CO_2_ (BioMedical Engineering Laboratory, Warsaw University of Technology, Warsaw, Poland). For spheroids, HCT116 cells (ATCC, Manassas, VA, USA) were cultured in DMEM (1 g/L glucose) supplemented with 10% fetal bovine serum, 2 mM glutamine, 50 μg/mL streptomycin, and 60 μg/mL penicillin. The cells were kept at 37 °C in a humidified atmosphere of 5% CO_2_. The cells were split twice a week and cell morphology and growth monitored on a weekly basis. Agarose-coated 96-well plates were obtained by preparing and autoclaving (121 °C, 20 min) a solution of agarose (Sigma-Aldrich Chemie GmbH, Darmstadt, Germany) in non-supplemented DMEM (1.5% *w*/*v*). The agarose solution was preheated in a water bath and 50 μL added to each well of a 96-well flat-bottom microtiter plate under sterile conditions. The plates were left to cool to room temperature and repacked. Plates could be stored at room temperature for 10 days.

### 2.7. In Vitro Cytotoxicity

Cytotoxicity and anticancer activity against MCF7, Caco2, and WI-38 cells were assessed as described previously [31]. We tested HAPs and HAP-Ps for cytotoxicity at 12.3, 37, 111, 333, and 1000 µg/mL. For all prepared nanoformulations and free Pip, anticancer activity was assessed at 2.4, 7.4, 22, 66, and 200 µg/mL. The concentrations of the nanoformulations were designed to obtain an equivalent amount of Pip in each. All stock solutions were prepared in PBS (Gibco/Life Technologies, Thermo Fisher Scientific, Langenselbold, Germany). PBS without nanoformulations or Pip was used as a control. Treated cells were incubated for 48 h and 72 h under the same conditions and the absorbance at 540 nm measured using a Robotnik P2000 ELISA reader (Robotnik India PVT LTD, Thane, India). Assays were performed in triplicate and the data expressed as mean ± standard deviation (SD) in terms of cell viability.

Cytotoxicity and anticancer activity against monolayer HCT116 cells were assessed by the XTT assay. The HCT116 cells (2 × 10^5^ cells/mL per well) were seeded in 96-well tissue culture plates. The cell monolayers were confluent after 24 h at 37 °C in a humidified atmosphere containing 5% CO_2_. The medium was removed and each well washed with PBS before adding suspensions of sample in culture medium at various concentrations. Each experimental plate contained replicates of negative control (NC, cells in pure medium), positive control (PC, cells treated with 2% Triton X-100), and blank control (BL, no cells). All cultures were incubated for 24, 48, and 72 h under the same conditions. We observed cell morphology under inverted light microscopy to detect the following morphological alterations: cell rounding and shrinking, loss of confluency, and/or cytoplasm granulation and vacuolization). We evaluated the cellular metabolic activity using the 2,3-bis-(2-methoxy-4-nitro-5-sulfophenyl)-2H-tetrazolium-5-carboxanilide (XTT) assay. The XTT reagent (Thermo Fisher, Eugene, OR, USA) solution (1 mg/mL) was prepared in culture medium and PMS (5 mM) added shortly before application to cells. Next, 50 µL of XTT solution with PMS in culture medium was added to each well and incubated at 37 °C in a humidified atmosphere containing 5% CO_2_ for 4 h. The plates were shaken gently for 5 min and the absorbance read using a BioTek reader at λ_1_ = 450 nm and λ_2_ = 630 nm. Each sample was run four times and the data expressed as mean ± standard deviation (SD) in terms of cell viability.

For the formation of HCT116 spheroids, 200 μL of HCT116 cell suspension with 5 × 10^3^ cells was seeded into each well of an agarose-coated 96-well plate. The plates were incubated at 37 °C in 5% CO_2_. Spheroids formed by day 4, and the diameter of the formed structures was measured using phase-contrast microscopy and Zeiss software (Axio Observer 3, Carl, Zeiss Microscopy GmbH, Germany). Each well was exchanged for 100 μL of fresh medium and sample suspension. HAPs and HAP-Ps were prepared at 12.3, 37, 111, 333, and 1000 µg/mL. Pip, HAP-Pip7.2, HAP-P-Pip7.2, HAP-Pip9.3, HAP-P-Pip9.3, HAP-Pip9.3-GA, HAP-P-Pip9.3-GA, HAP-Pip9.3-GA-FA, and HAP-P-Pip9.3-GA-FA were prepared in supplemented DMEM medium at 2× concentration to obtain the final concentration of 2.4, 7.4, 22, 66, or 200 μg/mL in each well. The suspensions were vortexed before addition to the wells. The 3-day-old spheroids were incubated with prepared suspensions for 48 h and 72 h before measuring cytotoxicity and diameter. The positive control was 10% Triton X-100 in the standard medium. The acid phosphatase assay (APH) was performed to evaluate cytotoxicity. The assay buffer was made up of 3M stock solution (pH 5.2), 0.1 M sodium acetate (Sigma-Aldrich Chemie GmbH, Schnelldorf, Germany), and 0.1% (*v*/*v*) Triton X-100 (Sigma-Aldrich Chemie GmbH, Schnelldorf, Germany) in deionized/distilled water. The buffer was stored at 4 °C for up to 4 weeks. Immediately before use, we prepared the substrate solution (final pH 4.8) by supplementing the assay buffer with 2 mg/mL ImmunoPure p-nitrophenyl phosphate (Sigma-Aldrich, Merck KGaA, Darmstadt, Germany). At each time point, the sphericity, spheroid integrity, and diameter were analyzed by phase-contrast imaging before the APH assay. Spheroids were carefully transferred with the entire supernatant into standard flat-bottom 96-well microplates using a manual eight-channel pipettor. The plates were washed with PBS 3× by carefully replacing 160 μL of the liquid above spheroids with PBS, leaving 100 μL of liquid in each well after the washing process. To each well, we added 100 μL of APH assay buffer. After incubating for 90 min at 37 °C, we added 10 μL of 1 N NaOH (Sigma-Aldrich Chemie GmbH, Germany) to each well. Within 10 min, we measured the absorption at 405 nm using an Epoch Biotek Microplate reader (Epoch Biotek, Winooski, VT, USA).

### 2.8. Anticancer Effects

In 24-well culture plates, HCT116 cells (5 × 10^5^ cells/well) were grown on sterile 12-mm-diameter glass coverslips to sub-confluence and allowed to attach for 24 h. For the cellular uptake study, the cells were treated with HAP, HAP-P, HAP-Pip9.3-GA, HAP-P-Pip9.3-GA, HAP-Pip9.3-GA-FA, and HAP-P-Pip9.3-GA-FA at 100 μg/mL in culture medium for 4, 24, 48, and 72 h. Cells without treatments were used as a control. The treated cells were washed with PBS before being fixed with 4% paraformaldehyde in PBS for 2 h at room temperature. The cells were then dehydrated in a graded ethanol series (30, 50, 70, and 98%; 30 min each) before drying in a laminar chamber overnight.

### 2.9. Statistical Analysis

Data are expressed as mean ± SD. Significant differences were calculated by analysis of variance. Means were compared by the least significant difference at *p* < 0.05. Cytotoxicity towards MCF7, Caco2, and WI-38 cells was evaluated by IRRE STST 2005 software (ERRI Institute). For monolayer HCT116 cells and HCT116 spheroids, two-way ANOVA with Tukey’s multiple comparisons at *p* < 0.05 was performed using Prism software (Prism, GraphPad, San Jose, CA, USA). The drug-loading content and EE were evaluated by one way ANOVA [44].

## 3. Results

### 3.1. Morphology, Size, and Zeta Potential Measurements

**Figure 1:**
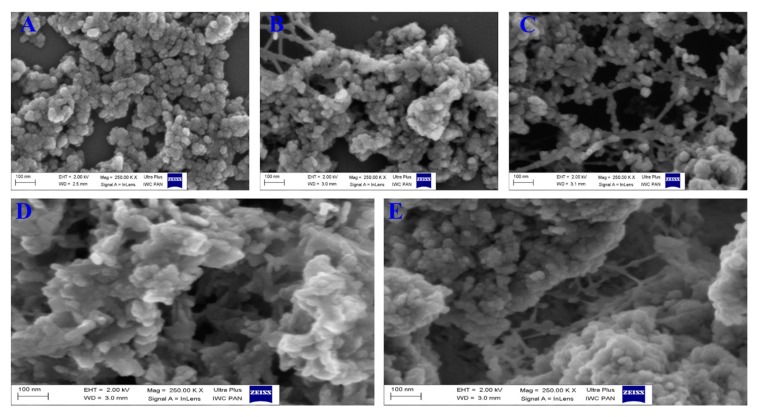
Field emission scanning electron microscopy (FE-SEM) at different stages. (**A**) Synthesized HAPs, (**B**) HAPs functionalized with phosphonate, (**C**) Pip loading, (**D**) GA coating, and (**E**) FA conjugation.

Figure 1 shows the morphological structure and differences before and after Pip loading, GA coating, and FA conjugation for HAPs. The nanoparticles nearly elongated to a spherical shape in aggregates (Figure 1A) due to the aggregative nature of hydroxyapatite materials at either the micro or nanoscale with various synthesis methods. The modification of HAPs with phosphonate led to some interconnection between the particles (Figure 1B). Further loading of Pip resulted in no change compared to HAP-P (Figure 1C) or HAP. Furthermore, a layer clearly formed (more white color seen) when GA was used to coat the loaded nanoparticles (HAP-P-Pip9.3-GA; Figure 1D). The same layer was observed after attaching FA to HAP-P-Pip9.3-GA to obtain HAP-P-Pip9.3-GA-FA (Figure 1E).

**Figure 2:**
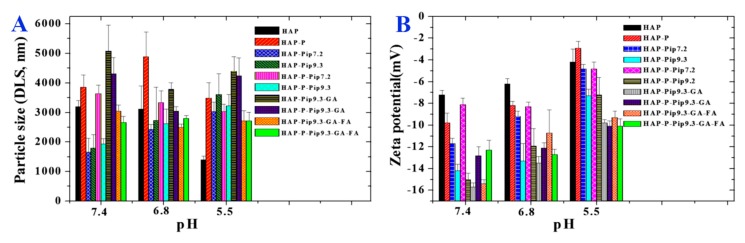
(**A**) Particle size measured by DLS for all prepared materials at different stages before and after loading, coating, and conjugation. (**B**) Zeta potential measurements in PBS under different pH conditions for all prepared materials at different stages before and after loading, coating, and conjugation.

Usually, HAPs in powder form feature aggregated nanoparticles, a cluster of nanoparticles unrelated to the synthesis method, leading to a larger size on the microscale when the HAPs are suspended in biological buffers, including PBS. We investigated the size of particles suspended in PBS adjusted to a pH of 7.4, 6.8, and 5.5 using DLS analysis. The size of some samples (Figure 2A) decreased or increased with changing pH; at acidic pH 5.5, the HAPs decreased in size (~1300 nm), but the size increased (~3000 nm) when the pH was neutral (7.4 or 6.8). In addition, the size of HAP-Pip9.3-GA greatly increased (~5000 nm) in neutral medium compared to acidic media. Nearly no change was seen in the size of HAP-Pip9.3-GA-FA and HAP-P-Pip9.3-GA-FA at various pH; the particle size was >2500 nm. Figure 2B shows that all samples had a negative zeta potential when suspended in PBS adjusted to neutral and acidic pH. The samples had less negative potential values at pH 5.5 compared to pH 7.4 or 6.8. Comparing the coated and non-coated nanoparticles, HAP-Pip9.3-GA, HAP-P-Pip9.3-GA, HAP-Pip9.3-GA-FA, and HAP-P-Pip9.3-GA-FA exhibited more negative zeta potentials than non-coated nanoparticles.

### 3.2. Specific Surface Area and Pore Size Distribution

**Figure 3:**
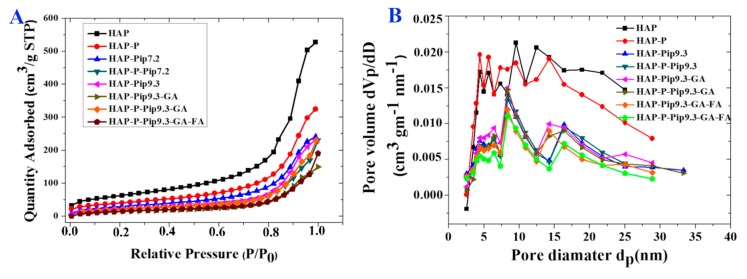
(**A**) The N_2_ adsorption isotherms for prepared materials at different stages before and after loading, coating, and conjugation. (**B**) The pore diameter distribution for prepared materials at different stages before and after loading, coating, and conjugation.

We measured the specific surface area by means of BET analysis (Figure 3A and Table 1). As expected, the specific surface area was diminished when particles were processed with different preparations compared to HAP and HAP-P. The specific area was decreased from 132.3 m^2^g^−1^ and 136.8 m^2^g^−1^ for HAP and HAP-P, respectively, to 70 m^2^g^−1^ and 61.6 m^2^g^−1^ for HAP-Pip9.3-GA-FA and HAP-P-Pip9.3-GA-FA, respectively. Table 1 also shows that the total pore volume property was highly affected by loading, GA coating, or FA conjugation, which was consistent with the surface area results.

The Barrett–Joyner–Halenda (BJH) method was used to characterize the pore size distribution and showed that the different preparations presented pores in the mesoporous range (Figure 3B and Table 1). The mean pore size ranged from 9 nm to 13 nm and was a little reduced following the loading and coating process, which is in agreement with the specific surface area and total pore volume results.

### 3.3. STA-DSC-MS Analysis

**Figure 4:**
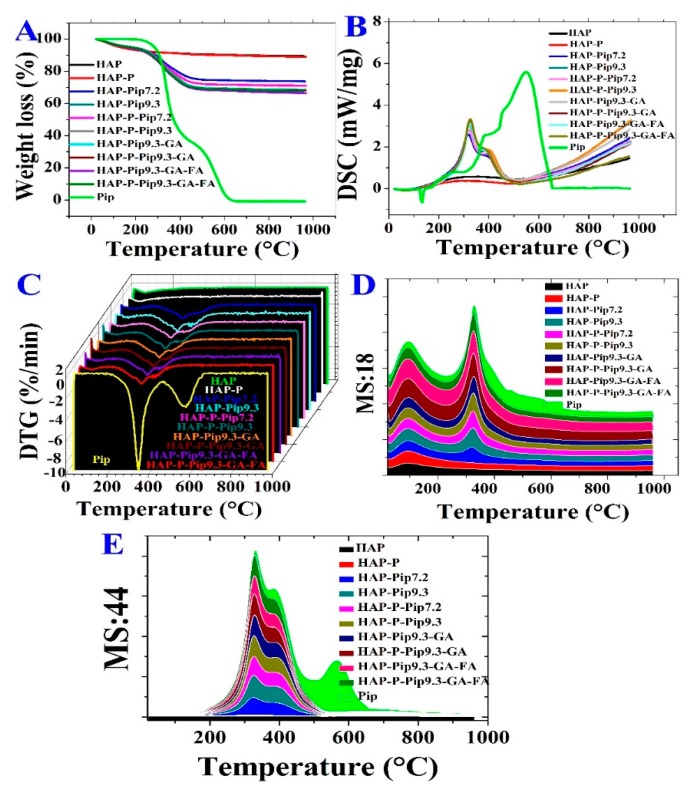
Simultaneous thermal analysis (STA) coupled with differential scanning calorimetry (DSC) and mass spectroscopy (MS) for all prepared materials at different stages before and after loading, coating, and conjugation. (**A**) Weight loss by STA, (**B**) DSC, and (**C**) DTG. (**D**,**E**) MS of water (**D**) and carbon dioxide (**E**) content.

The decomposition process of HAPs, Pip-loaded nanoparticles, and Pip occurred through different stages (Figure 4 and Table 1). All samples, except Pip, exhibited vaporization of moisture content from room temperature to 100 °C. The second stage of decomposition and weight loss started at 225 °C and ended at 416 °C for loaded materials compared to HAP and HAP-P (Figure 4A). A stable rate of the decomposition was achieved by the materials containing Pip, GA, and FA. The total Pip content decomposed through two stages starting at 243 °C and ending at 610 °C. The highest weight loss was recorded for materials containing GA and FA (HAP-Pip9.3-GA-FA, 33.5% and HAP-P-Pip9.3-GA-FA, 32.2%; Table 1).

We performed DSC to determine whether the crystallinity of Pip influences the loading process for the HAP and HAP-P. Figure 4B shows that Pip presented a sharp endothermic peak at 130 °C, which discloses the melting point and crystallinity. In contrast to Pip, all loaded materials exhibited no endothermic peak corresponding to Pip, revealing that the crystalline state changed to the amorphous state. In addition, a peak occurred in the range 200–500 °C for Pip-loaded nanoparticles, but not free Pip. The DTG thermograms (Figure 4C) show no peaks for HAP and HAP-Ps. Pip decomposed at 320 °C and 550 °C, but the loaded nanoparticles only decomposed at 320 °C.

To examine the evolved decomposition products in the gas state, we performed STA coupled to mass spectroscopy (STA-MS), monitoring the water content and CO_2_ as a qualitative determination. Regarding the moisture content of samples, two peaks were observed at 100 °C and 350 °C (Figure 4D) due to the MS:18 (water content) pattern for all Pip-loaded nanoparticles and free Pip, compared to HAP and HAP-P which did not have these peaks. An MS:44 (carbon dioxide content) pattern was detected with one peak centered at 320 °C (Figure 4E). As expected, CO_2_ was increased by increasing the organic content between all materials, especially free Pip, which also resulted in an additional small peak at 560 °C (green line). These results agree with the STA, DSC, and DTG results, indicating successful characterization and preparation.

**Figure 5:**
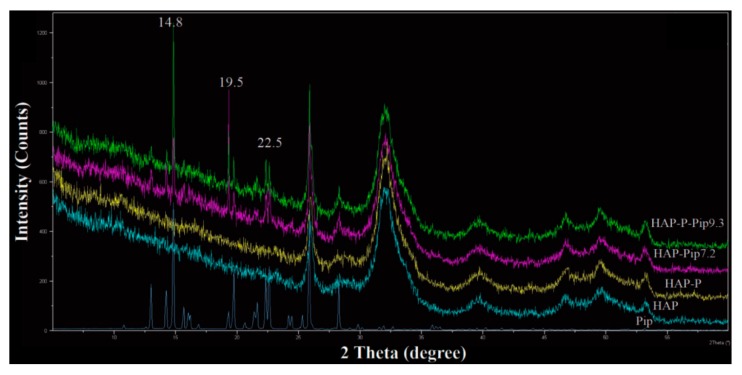
XRD patterns of select samples before and after Pip loading and of free Pip prodrug. The analysis was done based on the powder of prepared samples.

**Figure 6:**
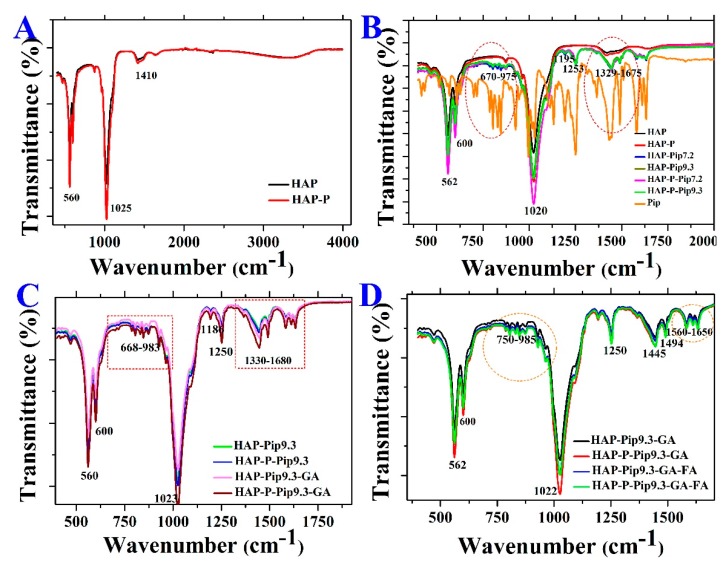
Fourier transform infrared spectroscopy (FTIR) spectra of HAPs before and after surface modification (**A**), after Pip loading at different pH compared to free Pip (**B**), before and after GA coating (**C**), and before and after FA conjugation (**D**).

Further characterization by XRD analysis showed that Pip loaded on either HAP or HAP-P resulted in some peaks corresponding to free Pip. The XRD patters showed three peaks, at 14.8°, 19.5°, and 22.5° (Figure 5), indicating that some Pip molecules are on the particle surface. To identify the functional groups on the surface of nanoparticles before and after modification, loading, and coating, we performed FTIR. The intensity of several peaks at 562, 600, 1020, and 1350 cm^−1^ was increased in HAP-P compared to HAPs (Figure 6A). The peaks at 562 and 600 cm^-1^ are attributed to the bending mode for P–O–P group, while peaks at 1020 and 1350 cm^−1^ correspond to phosphate stretching bands P–O [45]. By loading Pip on either HAP and HAP-P, several peaks were observed at 562, 600, 670–975, 1020, 1195, 1253, and 1329–1675 cm^−1^ (Figure 6B). The peaks at 670–975 cm^−1^ are attributed to C–H bending and C–O stretching; peaks at 1020 cm^−1^ could be account for symmetric =C–O–C stretching; peaks at 1195 cm^−1^ and 1253 cm^−1^ could be regarded to =C–O–C asymmetric stretching; and peaks ranging from 670–975 cm^−1^ could be corresponded to CH_2_ bending [46,47]. All these peaks were detected due to the presence of Pip in the nanoparticles. We found no differences in peak position for loaded HAP or loaded HAP-P. With the GA coating, several peaks had increased intensity and shifted at 560, 600, 668–983, 1023, 1186, 1250, and 1330–1680 cm^−1^ in HAP-Pip9.3-GA and HAP-P-Pip9.3-GA (Figure 6C), comparable to HAP-Pip9.3 and HAP-P-Pip9.3 (green and blue lines) seen close to one another, which confirms the coating of GA on the surface. The peaks at 900–1220 cm^−1^ could be regarded as fingerprints of carbohydrates, while peaks at 1330–1680 cm^−1^ could be due to COO- symmetric and asymmetric stretching [48,49]. Attaching FA (blue and green lines), the peaks at 562, 600, and 1022 cm^−1^ decreased, whereas the peaks at 750–985, 1250, 1445, 1494, and 1560–1650 cm^−1^ increased (Figure 6D), which discloses FA on the surface. The most important peaks are those that appeared at 1560–1650 cm^−1^ which are associated with forming amide I and amide II bands by means of the reaction between the amino groups (–NH_2_) of FA and carboxylic acid groups (–COOH) of GA [50]. Thus, the carboxylic groups are responsible for interaction with FA.

### 3.4. EDS Analysis

We used EDS analysis to identify the chemical composition for Pip loaded, GA coated, and FA conjugated nanoparticles (Figure S1, supporting information). Calcium, phosphor, and oxygen are the main elemental components of HAP nanoparticles for all samples: HAP-Pip9.3, HAP-P-Pip9.3, HAP-Pip9.3-GA, HAP-P-Pip9.3-GA, HAP-Pip9.3-GA-FA, and HAP-P-Pip9.3-GA-FA. Carbon was also detected because of the presence of organic compounds of Pip, GA, and FA. These results confirm that the elements detected are related the HAP, Pip, GA, FA, and no impurities was detected.

### 3.5. Pip Loading

Table 2 shows the total loading capacity (TLC) and EE determined by UV–vis and TG methods. For TLC, UV–vis showed no significant effect (*p* < 0.05) between any nanoparticles loaded with Pip under any condition. TG analysis showed that pH affects TLC, but that TLC not significantly differed with surface modification; HAP-Pip-9.3 had the maximum TLC (22.49 ± 2.97 and 20.31 ± 1.00% by UV–vis and TG, respectively). This can be explained in terms of the specific surface area, pore volume and pore size distribution being the main factors affecting TLC. These properties are higher for HAP comparing to HAP-P nanoparticles. This in the line with previous studies with mesoporous materials in drug delivery [51,52]. Because UV–vis is a more accurate method than TG, its results provided valuable information that the preparation steps did not influence the Pip content of HAPs. However, in general, EE was significantly affected by pH, surface modification, and coating. However, significant differences were not detected between some samples. The UV–vis method showed that EE significantly increased after conjugation with FA, which prevents Pip molecules from a quick release. The maximum EE was achieved with HAP-Pip9.3-GA-FA (85.05 ± 1.79%). The TG method showed no significant difference between GA and FA addition.

### 3.6. Solubility Measurements

The detailed solubility data for free Pip in various solvents and different pH are given in the Supplementary Information (Tables S1 and S2). The maximum solubility of Pip occurred at 72 h in ethanol (91.26 ± 3.47 µg/mL) compared to water/saline, 0.1 N HCL, and PBS adjusted to various pH values. The solubility of Pip was pH-dependent, increasing as pH decreased. Among other tested solvents, the solubility of Pip in PBS is important to provide valuable data for explaining the release of Pip from the developed DDS with HAPs. Pip solubility increased at pH 5 (36.0 ± 1.4 µg/mL) compared to pH 6.8, 7.4, and 9 (13.6 ± 3.4, 4.3 ± 0.8, and 0.2 ± 0.01 µg/mL, respectively). In regard to the mean solubility rate of Pip, the mean rate was ranked according to solvent as follows: ethanol > 0.1 N HCL > PBS > water/saline. Concerning the Pip rate in PBS at different pH, the results show that the rate increased with decreasing pH (0.50, 0.19, and 0.06 µg/mL/h at pH 5, 6.8, and 7.4, respectively) (Table S2).

**Figure 7:**
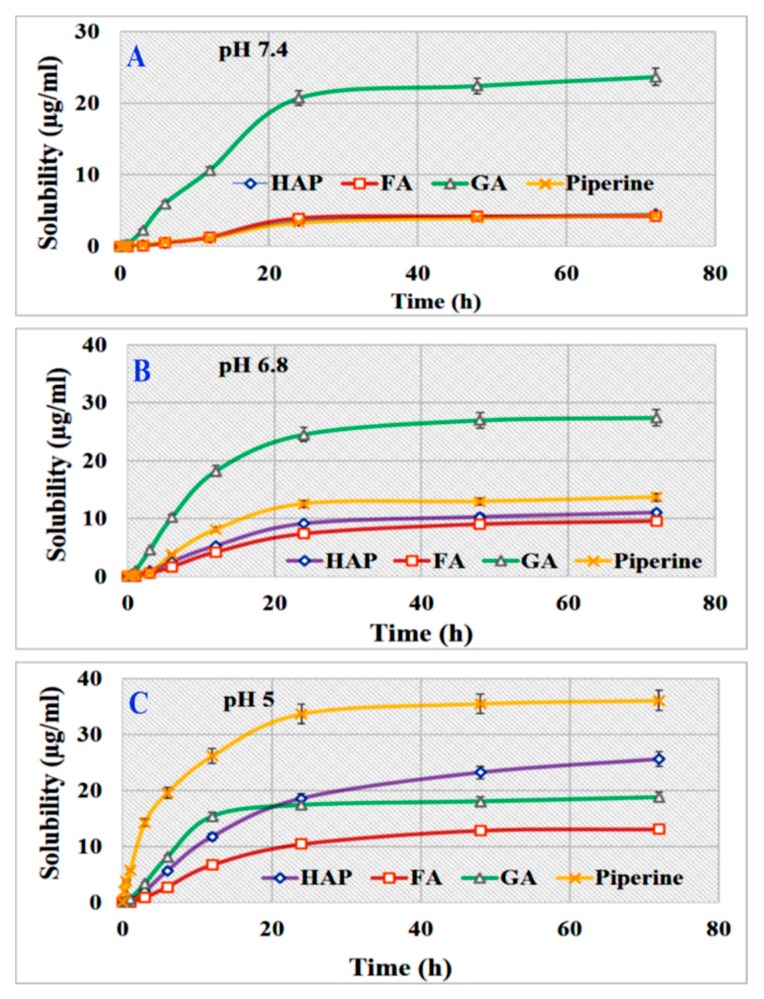
The solubility of Pip, HAP, GA, and FA in PBS at different pH. (**A**) pH 7.4, (**B**) pH 6.8, and (**C**) pH 5. Data is presented as mean ± SD.

The solubility data for HAPs, GA, and FA are shown in the supplementary information (Tables S3–S8 and Figures S2 and S3). The collective solubility data for Pip, HAPs, FA, and GA in different solvents are also given in the Supplementary Information. Here, we focused on the differences between their solubility in PBS as a function of pH. At pH 7.4, solubility ranked over 70 h was as follows: GA > Pip, HAPs, or FA (Figure 7A). Decreasing the pH to 6.8 resulted in GA > Pip > HAP > FA (Figure 7B), and the solubility of Pip increased at pH 5: Pip > HAP > GA > FA (Figure 7C). These results are important in light of the release effect, as GA would be the first to dissolve/degrade once the nanoparticle comes into contact with biological media at acidic or neutral pH. These results support Pip as being more soluble in acidic (pH 5 or 6.8) media and would be released inside the tumor cell upon degradation of the GA coating, which is the most soluble component at pH 6.8.

### 3.7. Effect of Loading Condition and Surface Modification on Release

Reviewing the release profile of HAP-Pip7.2 at different pH (Figure S4A), the fastest release of Pip in vitro was detected at 24 h at pH 5 (100.09 ± 0.32%). As the pH increased, the release decreased, reaching 101.68 ± 8.05% at pH 6.8 and 100.99 ± 1.77% at pH 7.4 after 36 and 48 h, respectively. Thus, Pip release requires a little longer time at pH 6.8 and 7.4 than pH 5. The same observation was made for the release of Pip from HAP-Pip9.3 (Figure S4B). Pip releases from HAP-Pip7.2 and HAP-Pip9.3 according to the Baker–Lonsdale kinetic model (Table S9). Comparing the RE data, we found no differences between the two nanoformulations. The MDT was more important for testing any formulation; for example, the MDT for Pip from HAP-Pip7.2 was 6.45 ± 0.01, 7.80 ± 0.01, and 9.87 ± 0.01 h at pH 5, 6.8, and 7.4, respectively. No differences were found between the two nanoformulations in terms of MDT. Thus, these data indicate that changing the pH of the loading process from 7.2 to 9.3 does not affect Pip release. 

The effect of surface modification by phosphate groups was determined by comparing the release profiles of HAP-P-Pip7.2 and HAP-P-Pip9.3 (Figure S4C,D and Table S9). Pip was released by HAP-P-Pip7.2 at 100.71 ± 3.68% (at 24 h), 100.02 ± 1.49% (at 36 h), and 103.76 ± 1.94% (48 h) at pH 5, 6.8, and 7.4, respectively. At pH 5 and 7.4, the nanoformulation released Pip according to the Baker–Lonsdale kinetic model. However, at pH 6.8, Pip was released according to the Korsmeyer–Peppas kinetic model. With HAP-P-Pip9.3, Pip reached its maximum release at 24 h at pH 5 (100.75 ± 2.75%), at 36 h at pH 6.8 (100.76 ± 8.69%), and at 48 h at pH 7.4 (100.91 ± 6.96%). For HAP-P-Pip7.2, the RE values at all pH values differed significantly from one another and were 74.89 ± 0.01%, 70.11 ± 0.07%, and 72.06 ± 0.09% at pH 7.4, 6.8, and 5, respectively. For HAP-P-Pip9.3, no difference was found between RE values at pH 7.4 (69.50 ± 0.06%), pH 6.8 (70.29 ± 1.08%), or pH 5 (69.42 ± 0.06%). Regarding the MDT values for HAP-P-Pip7.2, they were 12.06 ± 0.83 h, 10.76 ± 0.67 h, and 6.71 ± 0.24 h at pH 7.4, 6.8, and 5, respectively. For HAP-P-Pip9.3, the MDT values were 14.64 ± 1.41 h, 10.70 ± 1.00 h, and 7.34 ± 0.33 h at pH 7.4, 6.8, and 5, respectively. Overall, by comparing the release of Pip from HAP-Pip7.2 vs. HAP-P-Pip7.2, HAP-Pip9.3, and HAP-P-Pip9.3, we can conclude that there were no significant differences. The release of Pip reached 100%, with a longer time at pH 7.4 and fastest at pH 5. The different values for RE and MDT between Pip-loaded HAP or HAP-P shows the effects of surface modification on Pip release.

### 3.8. Effect of Coating on Release

**Figure 8:**
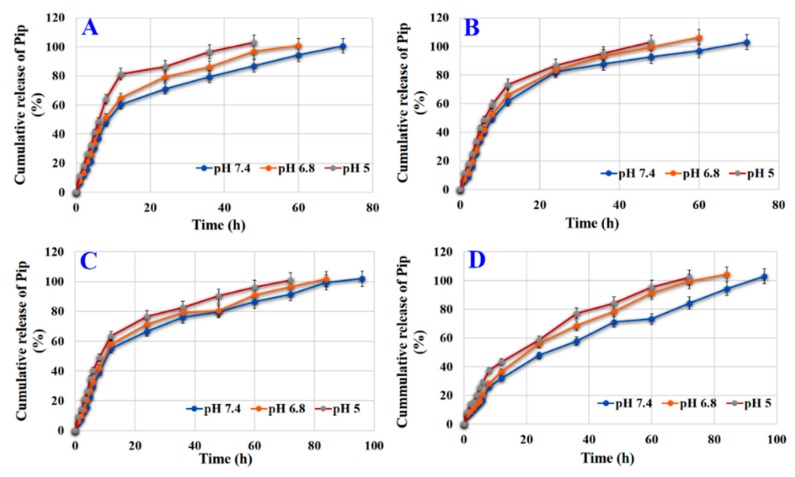
In Vitro release of Pip from PBS under different pH conditions. (**A**) HAP-Pip9.3-GA, (**B**) HAP-P-Pip9.3-GA, (**C**) HAP-Pip9.3-GA-FA, and (**D**) HAP-P-Pip9.3-GA-FA. Data is presented as mean ± SD.

Figure 8A and Table S9 show that HAP-Pip9.3-GA released Pip at 100.66 ± 4.5% (at 72 h), 100.78 ± 5.5% (at 60 h), and 102.88 ± 4.0% (at 48 h) at pH 7.4, 6.8, and 5, respectively, according to the Baker–Lonsdale kinetic model (Table S9). The RE values were 74.27 ± 0.0%, 76.47 ± 0.02%, and 80.01 ± 0.04% at pH 7.4, 6.8, and 5.5, respectively. The MDT values reached 18.53 ± 0.0, 14.12 ± 0.05, and 9.59 ± 1.03 h at pH 7.4, 6.8, and 5, respectively. 

For HAP-P-Pip9.3-GA, 100% of Pip was released differently based on pH; pH 7.4 took the longest time and pH 6.8 and 5.5 took a shorter time (Figure 8B and Table S9). Pip was released according to the Baker–Lonsdale kinetic model. The RE values were ~79%, showing no significant difference regarding pH changes. The MDT values increased at pH 7.4 (15 h) compared to pH 6.8 (12.4 h) and pH 5 (10.5 h). As shown in Figure 8C and Table S9, HAP-Pip9.3-GA-FA released 101.95 ± 7.02% (at 96 h), 101.61 ± 6.3% (at 84 h), and 100.98 ± 4.2% (72 h) Pip at pH 7.4, 6.8, and 5.5, respectively, according to Baker–Lonsdale kinetic model. RE values reached 75.62 ± 1.2%, 75.40 ± 1.4%, and 77.35 ± 1.2% at pH 7.4, 6.8, and 5, respectively. The MDT values were 23.41 ± 0.6, 20.67 ± 0.4, and 16.31 ± 0.1 h at pH 7.4, 6.8, and 5.5, respectively. HAP-P-Pip9.3-GA-FA released 100% after 96 (pH 7.4), 84 (pH 6.8), and 72 (5) h (Figure 8D and Table S9) according to the Korsmeyer–Peppas model. The RE values were 64.09 ± 0.4%, 69.02 ± 0.51%, and 68.94 ± 0.07% at pH 7.4, 6.8, and 5, respectively. The MDT values were higher at pH 7.4 (34.47 ± 3.2 h) than pH 6.8 (22.30 ± 0.9 h) and pH 5 (26.09 ± 1.0 h).

### 3.9. Cytotoxicity of HAPs

The cytotoxicity activity in terms of cell viability was investigated in several cancer cell lines. The results for MCF7, Caco2, and WI-38 cells are described in the Supplementary Information (Figure S5 and S6). Here, we discuss the promising results from HCT116 cells.

**Figure 9:**
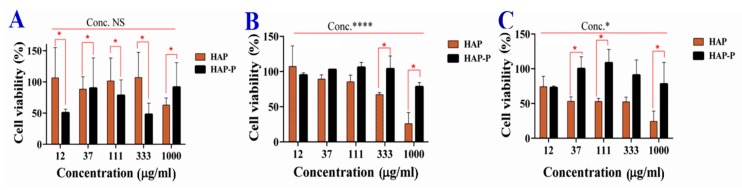
Cell viability of monolayer HCT116 colon cancer cells after treatment with HAPs a HAP-Ps for 24 h (**A**), 48 h (**B**), and 72 h (**C**). The red asterisk (*) indicates significant (*p* < 0.05) differences between HAPs and HAP-Ps at the same concentration. The black asterisk (*) indicates significant differences between concentrations. The black asterisk (****) indicate very high significant differences between concentrations. NS, not significant. All data are expressed as mean ± SD.

Figure 9 shows that the viability of HCT116 cells was significantly inhibited based on concentration, incubation time, and nanoparticle type. A strong reduction in viability was recorded at 72 h. After 24 h, no significant difference in cell viability was detected between the concentrations of HAP and HAP-P, but a significant effect was observed after 48 h and 72 h. A strong reduction in cell viability occurred when cells were treated at 1000 µg/mL. Regarding the effects of HAP and HAP-P on cytotoxicity, a significant difference was found after 48 h and 72 h, but not at 24 h. HAPs had more cytotoxicity than HAP-Ps towards HCT116 cells. Concerning the cytotoxicity of HCT116 spheroids, Table 3 shows no significant differences (*p* < 0.05) between HAP and HAP-P after 48 h and 72 h. However, viability was significantly decreased by increasing the concentration after 48 h. The viability reached 78.69 ± 6.9% (HAP) and 88.58 ± 5.0% (HAP-P) when the spheroids were treated at 1000 µg/mL.

### 3.10. In Vitro Anticancer Effects

**Figure 10:**
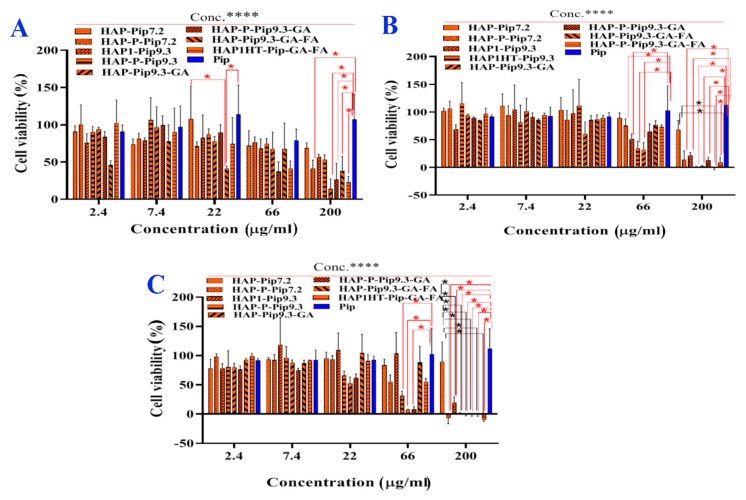
Cell viability (%) of monolayer HCT116 colon cancer cells in the presence of prepared nanoformulations and free Pip. The cells were treated at different concentrations and incubated for 24 h (**A**), 48 h (**B**), and 72 h (**C**). A red asterisk (*) indicates significant (*p* < 0.05) differences between nanoformulations and free Pip, whereas a black asterisk (*) indicates significant differences between nanoformulations. NS, not significant. A black asterisks (****) indicate very significant differences. All data are expressed as mean ± SD.

Figure 10 shows that the cell viability of monolayer HCT116 cells was significantly (*p* < 0.05) dependent on concentration, incubation time, and delivery method. Increasing the incubation time from 24 h to 72 h inhibited viability, with the strongest inhibition after 72 h (Figure 10C). Treatment of cells at high concentrations of nanoformulations (66 µg/mL and 200 µg/mL) led to full inhibition. Regarding the delivery route, there were significant differences in viability inhibition between nanoformulations and free Pip. As most anticancer effects occurred after 72 h, the treatment (200 µg/mL) of monolayer HCT116 cells with, HAP-Pip9.3, HAP-P-Pip9.3, HAP-Pip9.3-GA, HAP-P-Pip9.3-GA, HAP-Pip9.3-GA-FA, and HAP-P-Pip9.3-GA-FA highly inhibited ~100%, compared to ~0 and 10% by free Pip and HAP-P-Pip7.2. These results confirm that nanoformulations containing GA and FA are required for full inhibition of cancer cells. 

For spheroids (Table 4), viability was significantly affected by different treatments, but the concentration dependence was associated with incubation time. We observed that viability was highly decreased at 72 h compared to 48 h with high concentrations of 66 and 200 µg/mL compared to other concentrations. After 48 h, the lowest viability was 78.69 ± 6.9%, 88.58 ± 5.0/%, 90.5 ± 13.5% for HAP-Pip9.3, HAP-P-Pip9.3, and Pip when spheroids were treated at 200 µg/mL. After 72 h, HAP-Pip9.3-GA-FA and HAP-P-Pip9.3-GA-FA inhibited viability to 53.8 ± 8.3% and 54.2 ± 9.9.0% of spheroids when treated at 200 µg/mL compared to 125.2 ± 12.0% by Pip (no inhibition). These results show a role of targeted nanoformulations against spheroids.

### 3.11. Microscopic Observations

**Figure 11:**
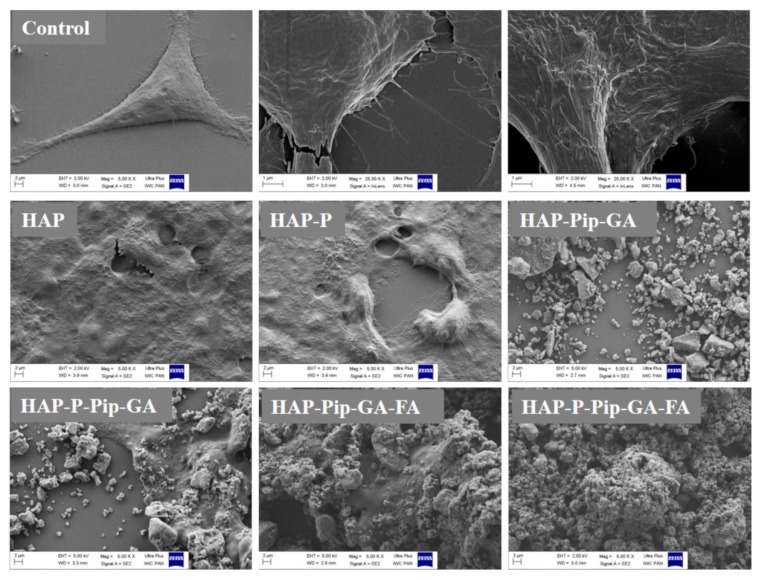
Anticancer effects in monolayer HCT116 colon cancer cells observed by field emission scanning electron microscopy (FE-SEM). The cells were treated with 200 µL of HAPs and HAP-Ps and select nanoformulations. Cells were incubated for 72 h. Untreated cells were used as controls and visualized at different magnifications, from 5 to 25 KX. Scale bar = 1 and 2 µm in control cells. Treated cells were visualized at a magnification of 5KX. Scale bar = 2 µm.

**Figure 12:**
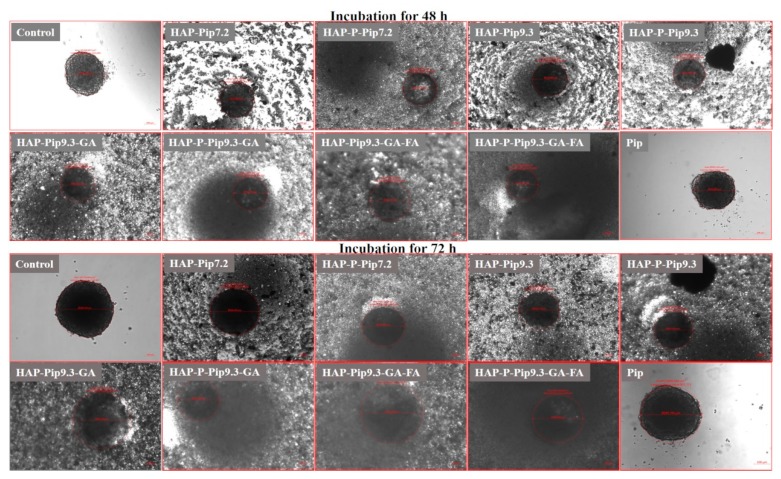
Anticancer effects in HCT116 colon cancer cell spheroids observed by inverted microscopy. The cells were treated with 200 µL of HAPs and HAP-Ps and select nanoformulations. Cells were incubated for 48 h and 72 h. Untreated cells were used as controls. Scale bars = 100 µm.

Using SEM, HAPs, and HAP-Ps did not change monolayer HCT116 cell morphology and viability compared to untreated control cells during all incubation periods (Figure 11 and Figures S7–S9). In contrast, HAP-Pip9.3-GA-FA and HAP-P-Pip9.3-GA-FA exhibited a strong visual anticancer effect after 72 h (Figure 11). At this time point, we observed numerous significant changes in cell morphology, including perforation of the cell membrane and disruption of intercellular adhesion, which indicates the efficient anticancer effect achieved in the viability assay (Figure 10C). These changes were also observed to some extent after 24 h and 48 h (Figures S8 and S9). Interestingly, HAP-Pip9.3-GA and HAP-P-Pip9.3-GA treatment resulted in perforation of the cell membrane and disruption of the intercellular adhesion after 48 h, and at 72 h these changes in cell morphology were high (Figure 11 and Figure S9). Inverted light microscopy observation found that free Pip had no influence on spheroid diameter compared to control cells. Shrinkage of the spheroids started after 48 h and 72 h in cells treated with HAP-Pip7.2, HAP-P-Pip7.2, HAP-Pip9.3, HAP-P-Pip9.3, HAP-Pip9.3-GA, HAP-P-Pip9.3-GA, HAP-Pip9.3-GA-FA, or HAP-P-Pip9.3-GA-FA compared to Pip-treated and control cells. After 72 h, the size of the spheroids was greatly decreased by treating with HAP-Pip9.3-GA, HAP-Pip9.3-GA-FA, or HAP-P-Pip9.3-GA-FA compared to free Pip and control (Figure 12).

### 3.12. Cellular Uptakes

**Figure 13:**
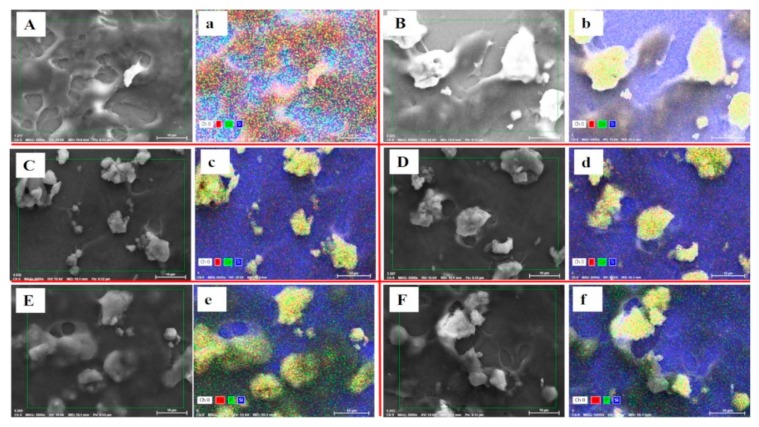
Cellular uptake observation by SEM and SEM-EDS elemental analysis mapping in monolayer HCT116 colon cancer cell treated at 100 µL and incubated for 48 h with HAPs (**A**,**a**), HAP-Ps (**B**,**b**), HAP-Pip9.3-GA (**C**,**c**), HAP-P-Pip9.3-GA (**D**,**d**), HAP-Pip9.3-GA-FA (**E**,**e**), and HAP-P-Pip9.3-GA-FA (**F**,**f**). Yellow color in elemental mapping images show the presence of both calcium and phosphorus (main components of HAP nanoparticles). Red dots correspond to calcium, and green dots to phosphor. Blue color is due to silicon present in the cover glass used for culture cells. Scale bar = 10 µm.

To confirm the cellular uptake of nanoformulations by cancer cells, we used the X-ray microanalysis technique, SEM-energy dispersive X-ray spectroscopy (SEM-EDS). This analysis allows us to confirm the presence of calcium and phosphorus, the main elemental composition of HAP nanoparticles, within the cells. Figure 13 shows internalization of HAP nanoparticles and nanoformulations in monolayer HCT116 cells after 48 h of incubation. It is worth noting that cellular uptake increased with conjugation of FA as in HAP-Pip9.3-GA-FA (e), which is reflected by the yellow color signaling Ca and P elements. These results confirm that FA facilitates nanoformulations active targeting through folate receptors-FA interaction. It leads to internalization in cancer cells, which agrees with the results of anticancer effects.

## 4. Discussion

Pip exerts anticancer effects via different mechanisms of action. Most of the mechanisms that have been reported are based on the inhibition of proliferation and survival as a result of the modulation of cell cycle progression, antioxidant activities due to detoxification of enzymes and suppression of stem cell self-renewal, and anti-apoptotic actions by many molecular signaling pathways [53]. Pip has been shown to play an important role in mediating several enzymes and transcription factors that contribute to hindering the process of invasion, metastasis, and angiogenesis during cancer progression [53,54,55]. Importantly, Pip is considered a potent inhibitor of p-glycoprotein (P-GP), which results in the inhibition of multidrug resistance in cancer [55]. Recently, Pip analogs have been developed to target P-GP and overcome drug resistance in cancer [56]. Interestingly, Pip has shown selective anticancer effects on cancer cells compared to normal cells [53]. Therefore, we intended to design a delivery system for Pip as a promising drug candidate derived from natural substances. We show for the first time that delivery of Pip-loaded HAPs using FA ligands for active targeting resulted in significant inhibition against colon cancer cells in monolayer or spheroids.

The HAPs exhibited an aggregation effect with a mix of nearly elongated to spherical shapes. The particle size of HAP powder was an average of 9.7 ± 0.1 nm based on the specific surface area measurements according to Kuśnieruk et al. [42]. SEM images demonstrated the obvious differences between particles before and after GA coating and FA conjugation, confirming the successful preparation. Upon the addition of HAP powder in the PBS adjusted to various pH, the nanoparticle size increased to micrometer-scale as expected based on the tendency of hydroxyapatite particles to agglomerate in biological media. These effects are related to their stability into aqueous media and zeta potential strength, as weaker potential values can induce electrostatic repulsion between particles, resulting in the induction of agglomeration [57]. HAPs at pH 7.4, 6.8, and 5.5 carry negative potential up to −15 mV. The plausible reason for negative zeta potential is OH groups [36]. Particles with zeta potential values < ±10 mV can agglomerate [58], leading to an increase in the size of particles when investigated by DLS. Thus, our results are consistent with previous reports [57,59]. We speculate that the main reason for the increase in HAP size is due to the agglomeration effect and not Pip loading or coating. After injection of the particles in vivo they are distributed in different organs depending on the different routes of in vivo application: sub-cutaneous, intramuscular, and intravenous injection [60,61]. Therefore, for future in vivo studies, the selection of intra-muscular, intradermal, or subcutaneous depot will be needed in order to avoid the barrier made by the reticulo-endothelial system (RES). Oral administration seems to be preferred, since Pip would be directly released in colon and targeted to cancerous cells.

The specific surface area, total pore volume, and pore size were greatly decreased when particles were loaded with Pip, coated with GA, and conjugated to FA compared to the starting material for both HAPs and HAP-Ps. This observation is expected and in agreement with previous studies [30,31,36]. Interestingly, the pore size distributions calculated by BET method from N2 adsorption/desorption measurements showed that HAPs had a pore size ranging from 9 nm to 13 nm. There are two possible explanations for this observation. First, the HAPs (10 nm) are mesoporous structures themselves. Second, the aggregation/agglomeration behavior of HAPs leads to free spaces between the particles. The second reason is more logical. This porosity is necessary for drug encapsulation, which decreases the surface area properties.

The thermal stability, drug content, crystalline, and decomposition characteristics of Pip can be explored by thermal analysis. STA showed that HAPs lost about 10 wt % as moisture up to 1000 °C, which in the line with data by Kuśnieruk et al. [42]. When HAPs were further processed by loading and coating, high percentages of weight loss were measured because of decomposition of the organic compounds Pip, GA, and FA. In this context, we can calculate the drug-loaded content in inorganic nanoparticles [62]. Pip shifted to the non-crystalline state, as the peak indicating its melting point was not observed in all loaded nanoparticles. This observation can be explained by most Pip molecules being entrapped inside pores on the surface of HAPs. These results are in agreement with reports of loading drugs in mesoporous nanoparticles [30,31,63]. Changing the crystalline state of a water-insoluble drug to the non-crystalline state using mesoporous materials can enhance solubility, increasing the drug therapeutic activity [64]. The DTG results confirmed the thermal stability and decomposition of Pip, GA, and FA. STA-MS revealed that no toxic substances are present in the materials. All thermal analysis results revealed a good relationship between each other (STA, DTG, DSC, MS) and showed their importance in complementary techniques for characterizing materials.

Using FT-IR analysis in the characterization of materials confirmed the Pip loading on HAPs, with several peaks at 562, 600, 670–975, 1020, 1195, 1253, and 1329–1675 cm^−1^ corresponding to free Pip, showing the fraction of Pip molecules on the surface. The appearance of shifted peaks increased peak intensities, and new peaks confirmed the GA coating and FA conjugation to loaded nanoparticles. Overall, the FT-IR results verified the successful preparation. Going further, the entrapment of Pip in pores changed to the non-crystalline state as confirmed by DSC and XRD.

The drug loading properties (TLC and EE) are the main factor determining drug delivery either long or short-term for desirable therapeutic actions. Previously, Shen et al. reported that a drug-loading content ≥10% is important [65]. Therefore, we determined these properties using two different techniques. The first was by thermal properties depending on weight loss data from STA. The total Pip content in HAPs and HAP-Ps ranged from 18 wt % to 20 wt % depending on the nanoformulation and pH. The EE reached 72–81wt % depending on the presence or absence of the GA coating and FA conjugation. The drug content calculated based on TG analysis was reported previously for inorganic nanoparticles used in drug delivery [31,62]. The second way was UV–vis spectroscopy, which is a commonly used method in most drug delivery strategies. The Pip content ranged from 16% to 22%, which differs from the TG calculation [62]. The EE determined by the UV–vis method reached 75–85%. We expect this difference to be due to the principle of both methods, as UV–vis depends on the absorbance of samples, whereas TG analysis depends on the thermal behavior of the carrier and drug. The UV–vis method seems to be more accurate than TG analysis. The Pip content of HAPs has not been reported previously, but our results are comparable with those of other strategies used for Pip loaded (4.7%) with mixed nano-sized micelles for cancer cell delivery [66]. The results prove the feasibility of HAPs as a Pip delivery vehicle.

The highest solubility of Pip is in ethanol compared to other solvents. The solubility of Pip in PBS is pH-dependent, and the highest solubility was achieved at pH 5 compared to pH 6.8 and 7.4. This reflects the shorter release time at pH 7.4. This solubility characteristic is attributed to its alkaloid structure. Alkaloids are known to be weak bases because of their oxygen and nitrogen termini, with easy solubility in strong acids, making them more stable. Alkaloids are hydrophobic because of the benzene ring and cyclic structures forming their main chemical skeleton. All of these factors make Pip soluble in an organic solvent (e.g., ethanol) and slightly soluble in water. The results align with the Pip solubility data available in the literature [67]. Another characteristic is the high rate of solubility at pH 5, which suggests that Pip is more stable, allowing diffusion in acidic release media with controlled-release behavior.

Importantly, the GA polymeric coating conjugated to FA prolongs the release effect because it hinders Pip release. The release parameters and kinetics were altered with surface modification and polymer coating of the drug-loaded nanoparticles. These results are similar to our previous results in mesoporous silica nanoparticles loaded with thymoquinone and coated with different polymer mixtures [31], as well as other studies [68]. Regarding the release of Pip from other DDSs, Shao et al. reported that Pip release from a self-emulsifying DDS was 80% over 1 h [29]. In another study, 100% of loaded Pip was lost from chitosan nanoparticles over 24 h [28]. Laha et al. showed that the release of Pip from electrospun cross-linked gelatin nanofibers was affected by the pH of the medium and >90% was released over 24 h, fitting to Higuchi kinetic model [69]. Thus, the current study demonstrates a promising prolonged release effect for Pip from agglomerated HAPs depending on the pH conditions.

Based on the solubility test for the components used in preparations, the GA would be the first to dissolve in contact with biological media of acidic or neutral pH. With respect to agglomerated HAPs, we propose that, in suitable release medium, HAP absorbs water and swells. Once it swells, HAP absorbs more water, leading to an increase in the nanoparticle pore size and subsequent release of Pip through these pores or the space between agglomerated nanoparticles. The process may continue until the HAP completely degrades [70].

The kinetic model of Pip releases changes from Baker–Lonsdale to Korsmeyer–Peppas for the nanoformulations tested at pH 6.8, HAP, and the coating composed of GA and FA. According to the literature, such kinetic models are characteristic of the release from inorganic or polymeric nanoparticles. However, a change in the in vivo pharmacokinetics would be expected when tested in animals [29]. The Baker–Lonsdale model is a modification of the Higuchi model. It was derived to describe drug release through an equation utilized for linearization of release data for micro/nano capsules or spheres while considering matrix porosity and pore size [71]. However, Korsmeyer–Peppas model tends to linearize release data through establishing an exponential relationship between drug release and time. This model is a semi-empirical equation describing drug release from polymeric nanosystems when its release mechanism is not known or when there is superposition of two or more apparently independent mechanisms of drug transport, relaxation, and diffusion within and from the particles [72]. On relating the presented information and the in vitro release data of Pip depending on HAP spherical to elongated shape, pore volume and size, as well as the polymers employed. Thus, when release of Pip from HAP nanoparticles is according to Baker–Lonsdale model, this would reveal that for such particles, pore size, volume and particle shape is the rate-determining factor over its polymeric coating. Consequently, when Korsmeyer–Peppas is the best fitting, the reverse is true.

The RE and MDT for Pip changed with surface modification and coating. Before coating, RE increased with increasing pH (pH 7.4 > pH of 6.8 > pH 5), whereas after coating, the RE increased with decreasing pH (pH 5 > pH 6.8 > pH 7.4). MDT values significantly increased with surface modification and coating, with greater increases as pH increased (pH 7.4 > pH 6.8 > pH 5). The maximum MDT was measured for HAP-P-Pip9.3-GA-FA. The addition of a polymeric coating forms a sheath around encapsulated Pip, resulting in a decreased RE at pH 7.4 but increased RE at pH 5. The reason for this finding is the inherent gelling property of GA. Gelation of GA occurs because of its oxidation, leading to the formation of highly dense cross-linking. Acting as a barrier, this gel layer retards the rate of Pip diffusion into the release medium. Thus, GA is highly recommended for slow release of water-insoluble drugs. The extent of release in vitro depends on the cross-linking density, as well as drug payload. Thus far, it appears a GA coating can enhance the colloidal stability of nanoparticles [73]. The RE and MDT are used as model-independent kinetic parameters that can provide insights on drug absorption. Release efficiency is the area under the dissolution curve up to certain time, (t), expressed as percentage of area of rectangle described by 100% dissolution at the selected time [74]. While MDT is kinetic parameter used to characterize drug release rate and indicate drug release retarding efficiency of polymers used. It depends on dose/solubility ratio [75].

The results indicate strong anticancer activity of Pip through targeted delivery compared to free Pip and with less cytotoxicity towards normal cells. These results are in line with data published previously on HT-29 colon cancer cells, in which cell killing was via an apoptotic mechanism [20]. The effect also depends on the cancer cell line, time, and concentration [53]. Targeted delivery through HAP-Pip9.3-GA-FA and HAP-Pip9.3-GA-FA results in full inhibition of monolayer HCT116 cells compared to other tested lines treated at 200 µg/mL, showing a selective anticancer effect compared to MC7 breast cancer and Caco2 colon cancer cells. Further exploring the anticancer effects in HCT116 spheroids disclosed a weaker effect of ~50%. The high activity of free Pip strongly inhibits monolayer HT-29 colon cancer cells compared to HT-29 spheroids [20]. These results agree with previous data on HT-29 spheroids showing that drug-loaded nanoparticles are more efficient than a free drug [76]. The main reason for the difference is that spheroids are a more complex structure, similar to the solid tumor structure. Thus, spheroids offer a more rational platform for screening anticancer effects than monolayer cells [76,77]. Thus, the 3D cell culture models, including spheroids, closely mimic the main characteristics of human solid tumors (e.g., organization of the structure, assembly of the cell layers, hypoxia, and nutrient gradients) [78]. Therefore, monolayer cancer cells poorly predict the therapeutic efficiency or outcome in in vivo animal studies [76]. Regarding spheroids, the preliminary results in the current study show that the strong effect can further improve anticancer effects with further developments and investigations.

HAPs and HAP-Ps exhibited no significant accumulation in cells compared to control cells, which is associated with their lack of ability to target cancer cells. Importantly, HAP-Pip9.3-GA-FA and HAP-P-Pip9.3-GA-FA had the strongest anticancer effects because of internalization in HCT116 cells compared to other formulations at 72 h. The significant changes in cell morphology were perforation of the cell membrane and disruption of intercellular adhesion, which indicates a high affinity of the FA-conjugated nanoparticles for the cell surface, probably due to the overexpression of folate receptors. This phenomenon substantially suppressed the viability of cells for reduced contact with the culture medium and nutrients, inhibiting intercellular contact, making cell division and contact with signal proteins difficult. Concerning the GA-coated nanoparticles, HAP-Pip9.3-GA and HAP-P-Pip9.3-GA also exhibited significant changes in cell morphology after 72 h related to the kinetic release data at low acidic conditions (see Figure 8). Drug release from HAPs is strongly stimulated by a pH drop [36]. The efficient cancer targeting by FA-conjugated agglomerated HAPs agree with previous studies of targeting colon cancer cells through FA conjugation of nanoparticles [79,80].

With spheroids, HAP-Pip7.2, HAP-P-Pip7.2, HAP-Pip9.3, HAP-P-Pip9.3, HAP-Pip9.3-GA, HAP-P-Pip9.3-GA, HAP-Pip9.3-GA-FA, and HAP-P-Pip9.3-GA-FA had visible changes compared to control and free Pip. The main changes were smaller diameter, shrinkage, and defragmentation of spheroids [81]. The nano-particulate-system containing FA is efficient for delivering Pip prodrug and accumulates in spheroids compared to free Pip; thus, anticancer effects are enhanced due to internalized of HAP-Pip9.3-GA-FA and HAP-P-Pip9.3-GA-FA in HCT116 cells spheroids compared to other formulations. This effect aggress with nanoparticles loaded with anticancer drug compared to free drug to target spheroids [76]. To summarize the results obtained from our study, Table 5 shows the findings for the targeted delivery proposed by HAPs compared to free Pip prodrug. The most anticancer effective nanoformulations in monolayer and spheroids are those with FA (HAP-Pip9.3-GA-FA 671 and HAP-P-Pip9.3-GA-FA). Our findings are in the line with using of nanomedicine application for cancer targeting strategies and controlled release of anticancer drugs by various nanostructures [82,83,84].

## 5. Conclusions

A novel anticancer targeted drug delivery nanoformulation consisting of aggregates of HAPs loaded with Pip was efficient against HCT116 colon cancer cells in vitro. We measured a high loading capacity of up to 22%. Longer release was observed at pH 7.4, whereas a higher release rate was obtained at pH 6.8 and pH 5. GA and FA coatings prolonged the release effect. Unloaded nanoparticles exhibited weak cytotoxicity towards WI-38 fibroblasts, even at high concentrations, and the synthetic HAPs are structurally identical to the hydroxyapatite found in human bones, which makes them suitable as a drug carrier. Full inhibition of monolayer HCT116 cells was achieved, and a weaker effect in MCF7 and Caco2 cells. The strong anticancer effect was confirmed by microscopic observation. Perforation of the cell membranes and disruption of intercellular adhesion was found. The results disclose that the released Pip align with the anticancer effects at 72 h, showing a sufficient release effect for Pip. Thus, the developed DDS for Pip shows potential for targeting HCT116 colon cancer cells.

## Figures and Tables

**Table 1:** Physicochemical properties of HAP before and after surface modification, Pip loading, coating, and conjugation.

Sample	S_BET_ (m^2^/g) ^a^	Total Pore Volume ^b^ (cm^3^/g)	Mean Pore Size Diamter ^c^ (nm)	Weight Loss % wt. ^d^	Weight Content % wt. ^e^
HAP	222 ± 3	0.815	12.8	10.69 ± 0.65	-
HAP-P	143 ± 3	0.501	11.6	11.46 ± 0.40	0.77 as P
HAP-Pip7.2	108 ± 3	0.372	-	28.78 ± 2.32	-
HAP-P-Pip7.2	78 ± 3	0.356	-	31.76 ± 1.01	-
HAP-Pip-9.3	83 ± 3	0.358	10.8	29.94 ± 0.89	-
HAP-P-Pip 9.3	81 ± 3	0.358	10.4	31.30 ± 0.31	-
HAP-Pip9.3-GA	56 ± 3	0.231	11.2	31.83 ± 0.61	1.89 as GA
HAP-P-Pip9.3-GA	79 ± 3	0.354	11.7	31.72 ± 0.62	0.42 as GA
HAP-Pip9.3-GA-FA	70 ± 3	0.165	9.4	32.01 ± 1.45	0.18 as FA
HAP-P-Pip9.3-GA-FA	61 ± 3	0.294	9.3	31.89 ± 1.16	0.17 as FA

^a^ Specific surface area measurement. ^b^ Pore volume from nitrogen adsorption measurements at 0.999 P/P°, ^c^ Mean size distribution based on the Brunauer–Emerett–Teller method, ^d,e^ Calculated from the thermogravimetric analysis.

**Table 2:** The total drug loading capacity (TLC) and entrapment efficiency (EE) of piperine using UV–vis and TGA methods.

Nanoformulation	TLC (%)		EE (%)	
	UV Method	TG Method	UV Method	TG Method
HAP-Pip7.2	16.82 ± 3.52 ^a^	18.08 ± 2.60 ^cd^	77.42 ± 3.2 ^ce^	72.33 ± 10.41 ^cd^
HAP-P-Pip7.2	20.14 ± 4.87 ^a^	18.71 ± 0.90 ^bd^	74.98 ± 2.60 ^e^	74.83 ± 3.61 ^bd^
HAP-Pip9.3	22.49 ± 2.97 ^a^	20.31 ± 1.00 ^a^	79.97 ± 0.85 ^bc^	81.23 ± 3.98 ^a^
HAP-P-Pip9.3	21.26 ± 3.17 ^a^	19.84 ± 0.59 ^a^	80.75 ± 3.33 ^e^	79.36 ± 2.36 ^a^
HAP-Pip9.3-GA	17.32 ± 5.49 ^a^	19.55 ± 0.59 ^ab^	77.90 ± 1.26 ^be^	78.20 ± 2.36 ^ab^
HAP-P-Pip9.3-GA	18.75 ± 1.33 ^a^	19.58 ± 0.59 ^ab^	76.58 ± 1.0 ^de^	78.32 ± 2.36 ^ab^
HAP-Pip9.3-GA-FA	17.27 ± 2.39 ^a^	18.15 ± 0.59 ^d^	85.05 ± 1.79 ^a^	72.60 ± 2.36 ^d^
HAP-P-Pip9.3-GA-FA	19.47 ± 1.20 ^a^	19.44 ± 0.59 ^abc^	78.61 ± 2.2 ^bcd^	77.76 ± 2.36 ^abc^

The letters (a,b,c,d, etc.) demonstrate the significant differences based on ANOVA analysis by means of lest significant differences (LSD) values at *p* < 0.05 between groups. The different letters show the significance, while the same letters show no significance.

**Table 3:** Cytotoxicity of HAPs and HAP-Ps on the cell viability (%) of HCT116 spheroids.

Concentration (µg/mL)	HAP	HAP-P
	48 h	
12 **	90.16 ±6.9	110.7 ±5.0
37 **	96.09 ±14.8	117.05 ±1.3
111 **	78.29 ±9.6	107.56 ± 12.0
333 **	88.18 ±6.4	91.74 ±7.0
1000 **	78.69 ±6.9	88.58 ±5.0
	**72 h**	
12	129.2 ±12.7	129.2 ±9.7
37	122.1 ±17.2	122.1 ±19.0
111	130.8 ±15.6	130.8 ±14.0
333	125.7 ±11.4	125.7 ±11.6
1000	120.7 ±6.7	120.7 ±4.3

** Significant difference (*p* = 0.0049) between concentrations at 48 h. No significant difference was observed between HAP and HAP-P at 48 or 72 h.

**Table 4:** Anticancer effect based on mean cell viability (%) in HCT116 spheroids.

Concentration (µg/mL)	Treatment								
	HAP-Pip7.2	HAP-P-Pip7.2	HAP-Pip9.3	HAP-P-Pip9.3	HAP-Pip9.3-GA	HAP-P-Pip9.3-GA	HAP-Pip9.3-GA-FA	HAP-P-Pip9.3-GA-FA	Pip
	**48 h**								
2.4	118.7 ± 14	170.4 ± 9	93.2 ± 10	116.5 ± 8	105.2 ± 8	106.6 ± 2	95.9 ± 5	78.4 ± 26	110.9 ± 8
7.4	110.9 ± 32	135.4 ± 17	85.4 ± 10	81.7 ± 9	112.2 ± 6	90.7 ± 19	104.8 ± 14	88.8 ± 33	113.0 ± 6
22	117.7 ± 25	153.3 ± 48	97.6 ± 16	103.2 ± 11	107.5 ± 9	95.2 ± 2	136.2 ± 14	79.1 ± 12	102.6 ± 11
66	112.0 ± 19	142.5 ± 19 *	104.8 ± 21	106.3 ± 14	114.5 ± 18	75.0 ± 4	155.2 ± 26 *	71.8 ± 14	102.8 ± 19 *
200	93.2 ± 17	141.7 ± 16 *	85.6 ± 11	86.0 ± 8	109.2 ± 10	105.2 ± 13	170.2 ± 19 *	107.5 ± 18 *	90.5 ± 13 *
	**72 h**								
2.4#	94.2 ± 8	87.6 ± 7	108.0 ± 15	121.0 ± 25	111.4 ± 17	108.4 ± 7	105.4 ± 6	103.9 ± 7	125.9 ± 11
7.4#	85.8 ± 4	86.9 ± 11	103.9 ± 4	104.9 ± 8	105.1 ± 53	106.7 ± 11	85.9 ± 7	105.2 ± 4	133.4 ± 9
22#	90.0 ± 12	88.1 ± 3	99.8 ± 12	99.3 ± 12	93.2 ± 4	106.7 ± 7	86.9 ± 12	100.7 ± 15	132.5 ± 12
66#	79.6 ± 30 *	83.0 ± 9 *	104.2 ± 23 *	98.8 ± 2 *	78.4 ± 7 *	106.0 ± 9 *	58.1 ± 15 *	89.4 ± 6 *	125.5 ± 19 *
200#	91.9 ± 6	67.4 ± 4 *	74.8 ± 13 *	70.3 ± 7 *	56.2 ± 6 *	78.1 ± 11 *	53.8 ± 8 *	54.2 ± 9 *	125.2 ± 12 *

We found a significant difference (*p* < 0.0001) between all treatments at 48 h and 72 h. # indicates significant difference (*p* < 0.0001) between concentrations at 72 h. * Significant difference between Pip and nanoformulation treatments at the same concentration (we selected 66 µL and 200 µL) due to multi-comparisons being difficult to present in a table.

**Table 5:** Summary of the results for various piperine (Pip) delivery methods.

Piperine Delivery Method	Structure	Solubility and Release Kinetics	Targeting Specifications
Pip (free form)	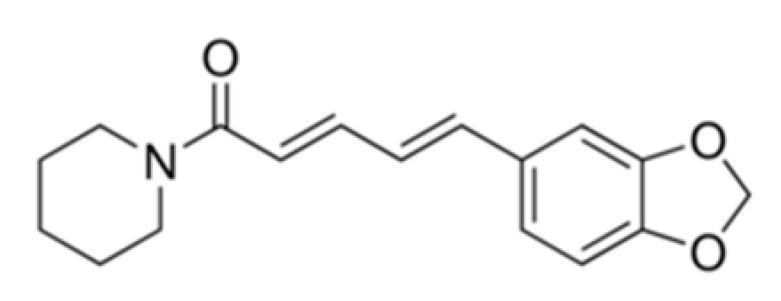	Higher solubility in PBS at acidic pH 5 than 6.8, 7.4, and 9.	Moderate cytotoxicity towards normal WI-38 cells. Weak activity against HCT116 colon cancer cells (monolayer and spheroids). Not effective. Non-targeting for colon.
HAP-Pip or HAP-P-Pip—Pip-loaded HAP not modified or modified with phosphonate. Loading at pH 7.2 and 9.3	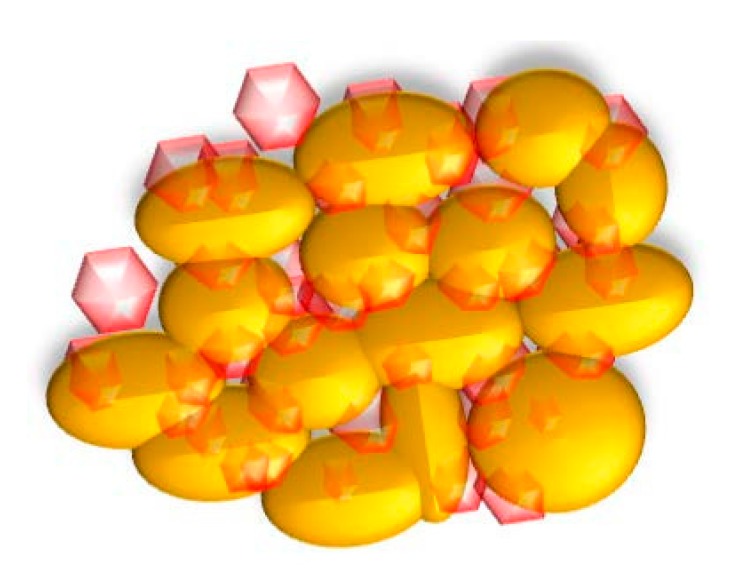	Short-term release. Release 100% of Pip capacity ~36 h (pH 6.8) and 24 h (pH 5).	Moderate cytotoxicity towards normal cells. High anticancer effect on monolayer colon cancer cells but not spheroids. Not enough cancer targeting. No change in cell morphology in monolayer or spheroids.
HAP-P-Pip9.3-GA-FA—Pip-loaded HAP modified with phosphonate, coated with gum Arabic, and conjugated to folic acid	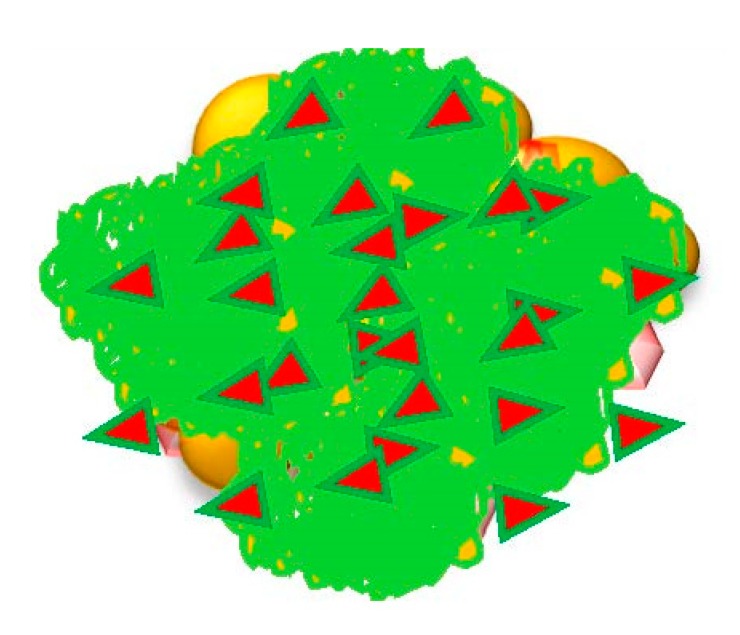	Long-term release. Release 100% of Pip capacity ~84 h (pH 6.8) and 72 h (pH 5).	Less cytotoxicity. High anticancer effect with full inhibition of monolayer HCT116 cells and ~60% inhibition for spheroids. High cancer-targeting of monolayer and spheroid HCT116 cells. Cancer targeting through folate receptors. Cell morphology changes: smaller size, shrinkage, and defragmentation of spheroids.

## Supplementary Materials

The following are available online at https://www.mdpi.com/1999-4923/12/1/70/s1, solubility study in details, calculations for total drug loading capacity and entrapment efficiency. Table S1. Solubility of piperine in solvents of various pH values; Table S2. Rate of piperine solubility in solvents of various pH values; Table S3. Solubility of hydroxyapatite in solutions of various pH values; Table S4. Rate of hydroxyapatite solubility in solutions of various pH values; Table S5. Solubility of folic acid in solutions of various pH values; Table S6. Rate of folic acid solubility in solutions of various pH values; Table S7. Solubility of gum Arabic in solutions of various pH values; Table S8. Rate of gum Arabic solubility in solutions of various pH values; Table S9. In-vitro release criteria of Pip from various HAP-NPs structures in PBS buffer media of different pH values; Figure S1. EDS analysis; Figure S2. Comparative solubility of piperine (Pip), hydroxyapatite (HAP), folic acid (FA), and gum Arabic (GA) in ethanol absolute; Figure S3. Comparative solubility of piperine (Pip), hydroxyapatite (HAP), folic acid (FA) and gum Arabic (GA) in 0.1 N HCl (pH 1.2); Figure S4. In vitro release Pip from PBS buffer under different pH conditions. Release profiles under pH 7.4, 6.8, and 5 for HAP-Pip7.2 (A), HAP-Pip9.3 (B), HAP-P-Pip7.2 (C), and HAP-P-Pip9.3 (D); Figure S5. Cytotoxicity evaluation of HAP and HAP-P on cancer and normal cell lines after 48 and 72 h of incubation; Figure S6. Cytotoxicity evaluation of all prepared materials and free Pip on cancer and normal cell lines after 48 and 72 h of incubation; Figure S7. The anticancer effects observation by field emission scanning electron microscopy (FE-SEM) in HCT116 colon cancer cells (monolayer) (4 h); Figure S8. The anticancer effects observation by field emission scanning electron microscopy (FE-SEM) in HCT116 colon cancer cells (monolayer) (24 h); Figure S9. The anticancer effects observation by field emission scanning electron microscopy (FE-SEM) in HCT116 colon cancer cells (monolayer) (48 h).
