# Supplementary material for: Effective Targeting of Colon Cancer Cells with Piperine Natural Anticancer Prodrug Using Functionalized Clusters of Hydroxyapatite Nanoparticles
Source: Pharmaceutics. 2020 Jan 16;12(1):70. doi: 10.3390/pharmaceutics12010070 (PMC7022489; doi:10.3390/pharmaceutics12010070)
Supplement: Supplementary file 1 [file pharmaceutics-12-00070-s001.pdf]

pharmaceutics

MDPI

Supplementary Materials: Effective Targeting of Colon Cancer Cells with Piperine Natural Anticancer Prodrug Using Functionalized Clusters of Hydroxyapatite Nanoparticles

Khaled AbouAitah, Agata Stefanek, Iman M. Higazy, Magdalena Janczewska, Anna Swiderska-Sroda, Agnieszka Chodara, Jacek Wojnarowicz, Urszula Szałaj, Samar A. Shahein, Ahmed M. Aboul-Enein, Faten Abou-Elella, Stanislaw Gierlotka, Tomasz Ciach, and Witold Lojkowski

1. EDS analysis

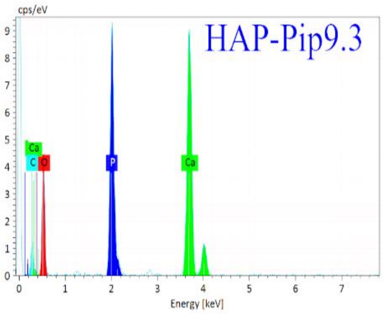

Element	At. No.	Netto	Mass [%]	Mass Norm. [%]	Atom [%]	abs. error [%] (1 sigma)	rel. error [%] (1 sigma)
Oxygen	8	3668	36.62	39.24	52.61	6.21	16.95
Calcium	20	14291	34.60	37.08	19.84	1.12	3.25
Phosphorus	15	11003	12.57	13.48	9.33	0.53	4.25
Carbon	6	884	9.52	10.20	18.22	2.39	25.06
		Sum	93.31	100.00	100.00		

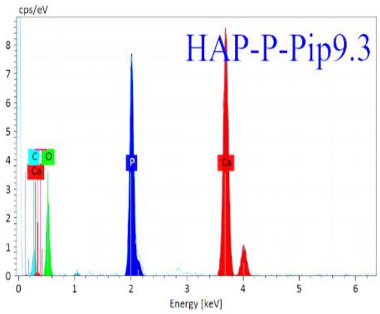

Element	At. No.	Netto	Mass [%]	Mass Norm. [%]	Atom [%]	abs. error [%] (1 sigma)	rel. error [%] (1 sigma)
Oxygen	8	4916	42.05	43.50	53.30	6.73	16.01
Calcium	20	12672	28.19	29.16	14.27	0.93	3.29
Carbon	6	1591	14.64	15.14	24.71	3.07	20.99
Phosphorus	15	10040	11.79	12.20	7.72	0.51	4.29
		Sum	96.67	100.00	100.00		

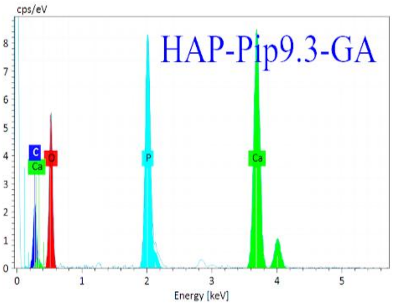

Element	At. No.	Netto	Mass [%]	Mass Norm. [%]	Atom [%]	abs. error [%] (1 sigma)	rel. error [%] (1 sigma)
Oxygen	8	4531	42.07	41.39	51.02	6.84	16.25
Calcium	20	13400	31.69	31.17	15.34	1.04	3.27
Carbon	6	1721	16.34	16.07	26.39	3.35	20.51
Phosphorus	15	10025	11.57	11.38	7.25	0.50	4.30
		Sum	101.66	100.00	100.00		

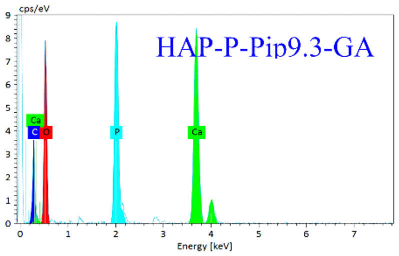

Element	At. No.	Netto	Mass [%]	Mass Norm. [%]	Atom [%]	abs. error [%] (1 sigma)	rel. error [%] (1 sigma)
Oxygen	8	5842	48.02	45.95	53.94	7.45	15.51
Calcium	20	11789	27.56	26.37	12.36	0.91	3.31
Carbon	6	2100	18.47	17.67	27.63	3.60	19.51
Phosphorus	15	9427	10.46	10.01	6.07	0.45	4.34
		Sum	104.50	100.00	100.00		

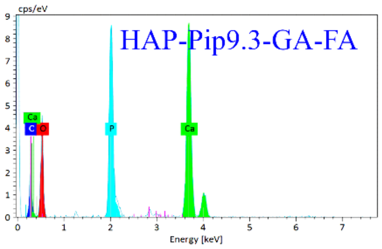

Element	At. No.	Netto	Mass [%]	Mass Norm. [%]	Atom [%]	abs. error [%] (1 sigma)	rel. error [%] (1 sigma)
Oxygen	8	3544	35.23	36.98	47.02	6.02	17.10
Calcium	20	13871	33.04	34.69	17.61	1.08	3.26
Carbon	6	1555	15.39	16.16	27.37	3.24	21.04
Phosphorus	15	10465	11.60	12.18	8.00	0.50	4.28
		Sum	95.27	100.00	100.00		

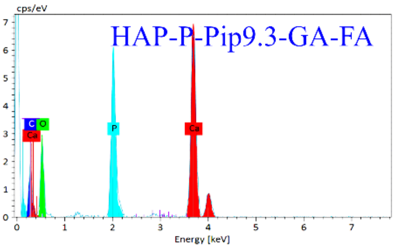

Element	At. No.	Netto	Mass [%]	Mass Norm. [%]	Atom [%]	abs. error [%] (1 sigma)	rel. error [%] (1 sigma)
Calcium	20	13588	30.96	35.00	17.48	1.01	3.27
Oxygen	8	3006	30.32	34.27	42.87	5.35	17.65
Carbon	6	1894	17.17	19.41	32.34	3.45	20.07
Phosphorus	15	9146	10.01	11.32	7.31	0.44	4.35
		Sum	88.45	100.00	100.00		

Figure S1. Energy Dispersive X-Ray (EDS) analysis for Pip loaded, GA coating, and FA conjugation HAP nanoparticles. The powder of samples paced on substate material and coated with gold-palladium before analysis. The carbon (C) content not accurate because the substrate has carbon. The analysis aiming to identify the chemical composition present in the nanoformulations.

2. Determination of total piperine loading capacity (TLC) and entrapment efficiency (EE)

2.1. UV-Vis method

Entrapment efficiency (EE)

To calculate piperine (Pip) entrapment efficiency, we first determined the Pip amount loaded in HAP particles according to Baspinar et al.[1] The loaded nanoparticles were dissolved in ethanol, followed by centrifugation for 30 min (at 2000 rpm and room temperature). Supernatant was collected by the centrifugation process (Table top cooling ultracentrifuge, Sigma 3-30KS, Sigma Laborzentrifugen GmbH, Germany) to measure the Pip concentration using a UV-vis spectrophotometer at respective λmax of Pip. The concentration was calculated using the prepared standard calibration curve of Pip in ethanol. Entrapment efficiency of Pip in nanoparticles was obtained indirectly according to the following equation:

$$EE(\%) = \frac{Initial\ amount\ of\ piperine\ "theoretical" - Amount\ of\ free\ piperine\ in\ supernatent}{Initial\ amount\ of\ piperine\ "theoretical"} X\ 100 \quad (1)$$

Determination of total loading capacity (TLC)

According to Li et l.[2]

Step 1: Experimental drug content (EDC)

Ethanol (5 ml) was used to dissolve 2 mg HAP loaded particles for proper extraction of Pip. The solution was stirred for 3 h for complete extraction of Pip in ethanol. The solution was then filtered. The filtrate was spectrophotometrically analyzed. And EDC was determined using the following formula:

$$EDC\ (\%) = \frac{Amount\ of\ piperine\ entrapped}{Total\ weight\ of\ nanoparticles} X100 \quad (2)$$

Step 2: Determination of total loading capacity (TLC)

Approximately 2 mg of Pip loaded HAP nanoparticles were added to 5 ml of ethanol and left under shaking for 24 h at room temperature. This was followed by centrifugation at 12000 rpm for 30 min (Table top cooling ultracentrifuge, Sigma 3-30KS, Sigma Laborzentrifugen GmbH, Germany). Pip content was determined in the separated supernatant. Pip was detected using UV spectrophotometry at the predetermined piperine λmax in ethanol standard solution. Total loading capacity was then determined according to the following equation:

$$Total\ Loading\ Capacity = \frac{Experimental\ Drug\ Content\ (EDC)}{Theoretical\ Content} X100 \quad (3)$$

Theoretical drug content was determined by calculation assuming that the entire drug present in piperine solution used got entrapped in HAP nanoparticles and no loss occurs at any stage of preparation.

2.2. TGA method

The calculation for TLC and EE was done using the data from thermal analysis and listed in Table 1 and 2.

Piperine total loading capacity % (TLC%) by TGA according to Equation (1)

TLC% = weight loss of piperine loaded materials with and without coating% − weight loss of nonmodified % (1). In Case of TLC for those having polymer, the obtained value of TLC – value of polymer %.

Piperine entrapment efficiency % (EE%) by TGA according to Equation (2)

Pip EE% = TLC /Expected theoretical loading content × 100

Expected theoretical loading content % (ETLC%) according to Equation (3) (the same Eq used for UV-Vis method)

ETLC% = (Weight of drug added (mg)/Weight of HAP and drug added (mg)) × 100.

3. Solubility study

The investigation was done by means of spectrophotometric analysis.

3.1. Solubility experiment protocol.

Excess amounts of piperine were weighed and added to closed glass vials containing 5 ml of each of the vehicles under test. Samples were preheated in a shaking water-bath at 37 ± 0.5 °C and 150 rpm, then left in the shaking water-bath for 3 days at room temperature until equilibrium. Supersaturated samples were then centrifuged at 5000 rpm for 10 minutes at room temperature to separate undissolved piperine. The supernatant was separated by centrifuge and filtered through a Millipore®0.45 μm membrane filter pre-rinsed with pre-prepared saturated piperine solution to reduce sorption of the solute on the used filter.[3] After that, ethanol was drop-wised to the turbid supernatant filtrate until the clear solution was obtained- this is to assure the dissolution of any residual piperine. The ethanol was removed from solution by evaporating for overnight under room temperature condition, then 2 ml (of filtrate solution) was used to analyze the piperine content using the UV-Vis spectrometer. The solubility measurements were repeated thrice.[4]

Solubility of hydroxyapatite, folic acid, Gum Arabic and piperine in ethanol, 0.1N HCl (pH 1.2), and phosphate buffer solutions of different pH values (pH 5.5, 6.8 and 7.4) were also examined, using the same experimental technique described: Higuchi and Connors standardized shake-flask method (1965).[5]

3.2. Results and Discussion of the solubility

Piperine

Solubility data of piperine in various solvents and different pH values would provide insights about the optimum release conditions and expected piperine kinetics in each media. On reviewing the presented data (Table S1), after 72 h, maximum solubility of piperine was noticed in ethanol (91.26 ± 3.47 μg/ml), while minimum solubility was detected in pH 9 buffer solution (0.286 ± 0.01 μg/ml). Solubility of piperine appeared to be pH dependent. As pH of solvent increases, solubility of piperine significantly decreases and vice versa ($p \geq 0.05$): solubility in 0.1N HCl (65.14 ± 3.99 μg/ml) > pH 5.5 solution (36.08 ± 1.44 μg/ml) > pH 6.8 (13.68 ± 3.41 μg/ml) > pH 7.0 (7.488 ± 1.54 μg/ml) > pH 7.4 (4.342 ± 0.82 μg/ml).

Mean rate of solubility of piperine was observed in the following different order: Ethanol (8.88 ± 1.00 μg/ml/h) > pH 1.2 (6.79 ± 0.98 μg/ml/h) > pH 5.5 (3.41 ± 0.54 μg/ml/h) > pH 6.8 (0.35 ± 0.01 μg/ml/h) > pH 7.0 (0.19 ± 0.01 μg/ml/h) > pH 7.4 (0.09 ± 0.00 μg/ml/h) > pH 9.0 (0.03 ± 0.00 μg/ml/h).

Rate of piperine solubility (Table S2) gives insights about the solvent ability to solubilize piperine. From the results obtained ethanol is efficient solvent to solubilize piperine compared other. The solubility rate was ethanol > 0.1N HCl > PBS (pH 5) > PBS (pH 6.8) > PBS (pH 7.4) > PBS (pH 9).

Table S1. Solubility of piperine in solvents of various pH values.

Time	Mean Solubility (μg/ml) ± SD						
(h)	Ethanol	pH 1.2	pH 5	pH 6.8	pH 7.0	pH 7.4	pH 9
0	0.00 ± 0.00	0.00 ± 0.00	0.00 ± 0.00	0.00 ± 0.00	0.00 ± 0.00	0.00 ± 0.00	0.00 ± 0.00
0.25	6.81 ± 0.26	4.98 ± 0.06	1.96 ± 0.04	0.09 ± 0.00	0.05 ± 0.00	0.02 ± 0.00	0.02 ± 0.00
0.5	8.55 ± 0.58	6.59 ± 0.04	3.84 ± 0.15	0.146 ± 0.01	0.08 ± 0.00	0.06 ± 0.00	0.03 ± 0.00
1	14.73 ± 0.44	11.23 ± 0.09	5.72 ± 0.94	0.322 ± 0.01	0.204 ± 0.01	0.11 ± 0.01	0.07 ± 0.00
3	28.45 ± 2.10	25.15 ± 1.02	14.29 ± 2.26	0.593 ± 0.02	0.413 ± 0.02	0.178 ± 0.01	0.123 ± 0.01
6	43.21 ± 3.52	38.08 ± 2.04	19.56 ± 3.38	3.742 ± 0.05	1.752 ± 0.06	0.593 ± 0.02	0.141 ± 0.01
12	74.97 ± 4.53	50.17 ± 3.05	26.13 ± 3.18	8.11 ± 1.24	4.361 ± 0.29	1.199 ± 0.07	0.175 ± 0.01
24	86.17 ± 4.32	57.62 ± 3.29	33.68 ± 1.32	12.55 ± 2.96	7.217 ± 0.85	3.301 ± 0.16	0.287 ± 0.01
48	90.59 ± 2.94	63.9 ± 4.11	35.51 ± 1.65	12.94 ± 3.27	7.301 ± 1.02	3.982 ± 0.59	0.288 ± 0.02
72	91.26 ± 3.47	65.14 ± 3.99	36.08 ± 1.44	13.68 ± 3.41	7.488 ± 1.54	4.342 ± 0.82	0.286 ± 0.01

Table S2. Rate of piperine solubility in solvents of various pH values.

Time	Rate of Piperine Solubility (μg/ml/h)						
(h)	Ethanol	pH 1.2	pH 5	pH 6.8	pH 7.0	pH 7.4	pH 9
0	0.000	0.000	0.000	0.000	0.000	0.000	0.000
0.25	27.240	19.920	7.840	0.360	0.200	0.080	0.080

0.5	17.100	13.180	7.680	0.292	0.160	0.120	0.060
1	14.730	11.230	5.720	0.322	0.204	0.110	0.070
3	9.483	8.383	4.763	0.198	0.138	0.059	0.041
6	7.202	6.347	3.260	0.624	0.292	0.099	0.024
12	6.248	4.181	2.178	0.676	0.363	0.099	0.015
24	3.590	2.401	1.403	0.523	0.301	0.138	0.012
48	1.887	1.331	0.739	0.269	0.152	0.083	0.006
72	1.268	0.905	0.501	0.190	0.104	0.060	0.004
Mean Rate of Solubility (µg/ml/h) ± SD	8.875 ± 1.002	6.788 ± 0.978	3.409 ± 0.541	0.345 ± 0.013	0.191 ± 0.010	0.085 ± 0.000	0.031 ± 0.000

Hydroxyapatite

The hydroxyapatite is chemically known as calcium hydroxyphosphate giving basic nature that leads to enhance its solubility in acidic media.[6] This aligns with the results presented in Table S3, where solubility increases as acidity increases. We can note that hydroxyapatite is highly soluble in 0.1N HCl (pH 1.2): 40.12 ± 2.16 µg/ml, whereas the minimum solubility was observed in pH 7.4. Hydroxyapatite showed low solubility in ethanol (2.48 ± 0.15 µg/ml) which is associated with its nature as a weak base inorganic material. The results show that the rate of solubility (Table S4) accords with the solubility results.

Table S3. Solubility of hydroxyapatite in solutions of various pH values.

**Time (h)**	**Mean Solubility (µg/ml) ± SD**				
	**Ethanol**	**pH 1.2**	**pH 5**	**pH 6.8**	**pH 7.4**
0	0.00 ± 0.00	0.00 ± 0.00	0.00 ± 0.00	0.00 ± 0.00	0.00 ± 0.00
0.25	0.00 ± 0.00	0.07 ± 0.01	0.03 ± 0.00	0.01 ± 0.00	0.00 ± 0.00
0.5	0.00 ± 0.00	0.22 ± 0.01	0.09 ± 0.01	0.05 ± 0.00	0.02 ± 0.00
1	0.01 ± 0.00	1.72 ± 0.08	0.42 ± 0.01	0.18 ± 0.01	0.08 ± 0.01
3	0.04 ± 0.00	6.88 ± 0.12	1.98 ± 0.05	0.93 ± 0.02	0.21 ± 0.01
6	0.10 ± 0.01	13.51 ± 1.19	5.63 ± 0.62	2.55 ± 0.16	0.45 ± 0.00
12	0.94 ± 0.07	21.52 ± 3.03	11.74 ± 1.44	5.29 ± 0.80	1.13 ± 0.05
24	1.66 ± 0.09	33.90 ± 2.55	18.57 ± 2.06	9.11 ± 1.03	3.52 ± 0.26
48	2.15 ± 0.11	38.14 ± 1.30	23.21 ± 1.99	10.26 ± 0.59	4.19 ± 0.99
72	2.48 ± 0.15	40.12 ± 2.16	25.60 ± 1.13	11.00 ± 0.76	4.58 ± 0.72

Table S4. Rate of hydroxyapatite solubility in solutions of various pH values.

**Time (h)**	**Rate of hydroxyapatite Solubility (µg/ml/h)**				
	**Ethanol**	**pH 1.2**	**pH 5**	**pH 6.8**	**pH 7.4**
0	0.000	0.000	0.000	0.000	0.000
0.25	0.000	0.280	0.120	0.040	0.000
0.5	0.000	0.440	0.180	0.100	0.04
1	0.010	1.720	0.420	0.180	0.08
3	0.013	2.293	0.660	0.310	0.07
6	0.017	2.252	0.938	0.425	0.075
12	0.078	1.793	0.978	0.441	0.094
24	0.069	1.413	0.774	0.380	0.147
48	0.045	0.795	0.484	0.214	0.087
72	0.034	0.557	0.356	0.153	0.064
Mean Rate of Solubility (µg/ml/h) ± SD	0.027±0.001	1.154±0.008	0.491±0.013	0.224±0.05	0.066±0.018

Folic acid

Folic acid is chemically a weak acid since it has acidic and basic terminals. [7] Thus, it is expected to be soluble, a weak acid and a strong base. The pKa value of FA is 8.26 reflecting its solubility in pH medium between pH 5 and pH 7, along with strong bases.[8] The results presented in Table S5&S6 confirm the listed data. We observed maximum solubility of FA at pH 5 (13.10 ± 0.90 µg/ml), ($p \geq 0.05$), followed by its solubility in pH 6.8 medium (9.55 ± 1.54 µg/ml), whereas the minimum solubility was at pH 1.2 and pH 7.4. Although, ethanol has a pH value between 7 and 8, however; it can solubilize FA, (7.19 ± 0.57 µg/ml), because of its organic nature.

Table S5. Solubility of folic acid in solutions of various pH values.

**Time**	**Mean Solubility (µg/ml) ± SD**				
**(h)**	**Ethanol**	**pH 1.2**	**pH 5**	**pH 6.8**	**pH 7.4**
0	0.00 ± 0.00	0.00 ± 0.00	0.00 ± 0.00	0.00 ± 0.00	0.00 ± 0.00
0.25	0.00 ± 0.00	0.00 ± 0.00	0.01 ± 0.00	0.00 ± 0.00	0.00 ± 0.00
0.5	0.01 ± 0.00	0.00 ± 0.00	0.03 ± 0.00	0.01 ± 0.00	0.01 ± 0.00
1	0.02 ± 0.00	0.02 ± 0.00	0.10 ± 0.00	0.04 ± 0.00	0.01 ± 0.00
3	0.13 ± 0.00	0.06 ± 0.00	0.83 ± 0.02	0.52 ± 0.01	0.05 ± 0.00
6	0.97 ± 0.03	0.19 ± 0.01	2.74 ± 0.06	1.63 ± 0.03	0.48 ± 0.01
12	2.66 ± 0.21	0.68 ± 0.01	6.59 ± 0.35	4.21 ± 0.16	1.28 ± 0.03
24	5.49 ± 0.42	0.98 ± 0.02	10.42 ± 1.03	7.37 ± 0.80	3.91 ± 0.11
48	7.15 ± 0.29	1.84 ± 0.09	12.87 ± 1.69	9.02 ± 0.73	4.16 ± 0.52
72	7.19 ± 0.57	2.06 ± 0.05	13.10 ± 0.90	9.55 ± 1.54	4.17 ± 0.33

Table S6. Rate of folic acid solubility in solutions of various pH values.

**Time**	**Rate of folic acid Solubility (µg/ml/h)**				
**(h)**	**Ethanol**	**pH 1.2**	**pH 5**	**pH 6.8**	**pH 7.4**
0	0.000	0.000	0.000	0.000	0.000
0.25	0.000	0.000	0.040	0.000	0.000
0.5	0.020	0.000	0.060	0.020	0.020
1	0.020	0.020	0.100	0.040	0.010
3	0.043	0.020	0.277	0.173	0.017
6	0.162	0.032	0.457	0.276	0.080
12	0.222	0.057	0.549	0.351	0.107
24	0.229	0.041	0.434	0.307	0.163
48	0.149	0.038	0.268	0.188	0.087
72	0.099	0.029	0.182	0.133	0.058
Mean Rate of Solubility (µg/ml/h) ± SD	0.094 ± 0.001	0.024 ± 0.001	0.237 ± 0.020	0.148 ± 0.001	0.054 ± 0.018

Gum Arabic

From data presented in **Tables S7 &S8** gum arabic showed maximum solubility in pH 6.8 (27.42 ± 3.06 µg/ml), followed by pH 7.4 (23.64 ± 1.85 µg/ml) then pH 5.5 (18.79 ± 1.57 µg/ml). However, its minimum solubility was in 0.1N HCl (0.513 ± 0.01 µg/ml) and finally, absolute ethanol (0.301 ± 0.02 µg/ml).

Table s7. Solubility of gum Arabic in solutions of various pH values.

**Time**	**Mean Solubility (µg/ml) ± SD**				
**(h)**	**Ethanol**	**pH 1.2**	**pH 5**	**pH 6.8**	**pH 7.4**
0	0.00 ± 0.00	0.00 ± 0.00	0.00 ± 0.00	0.00 ± 0.00	0.00 ± 0.00
0.25	0.00 ± 0.00	0.00 ± 0.00	0.09 ± 0.00	0.14 ± 0.00	0.12 ± 0.00
0.5	0.00 ± 0.00	0.00 ± 0.00	0.23 ± 0.01	0.36 ± 0.010	0.19 ± 0.00
1	0.00 ± 0.00	0.00 ± 0.00	0.55 ± 0.01	0.91 ± 0.02	0.35 ± 0.01
3	0.01 ± 0.00	0.02 ± 0.00	3.40 ± 0.02	4.52 ± 0.16	2.27 ± 0.01

6	0.03 ± 0.00	0.05 ± 0.01	8.11 ± 0.09	10.18 ± 0.32	5.99 ± 0.25
12	0.07 ± 0.01	0.110 ± 0.01	15.39 ± 2.30	18.22 ± 1.59	10.61 ± 0.99
24	0.121 ± 0.01	0.304 ± 0.00	17.41 ± 1.78	24.53 ± 2.80	20.70 ± 2.74
48	0.228 ± 0.01	0.488 ± 0.02	18.05 ± 3.42	26.98 ± 1.88	22.39 ± 3.16
72	0.301 ± 0.02	0.513 ± 0.01	18.79 ± 1.57	27.42 ± 3.06	23.64 ± 1.85

Table S8. Rate of gum Arabic solubility in solutions of various pH values.

**Time**	**Rate of gum Arabica Solubility (µg/ml/h)**				
**(h)**	**Ethanol**	**pH 1.2**	**pH 5**	**pH 6.8**	**pH 7.4**
0	0.000	0.000	0.000	0.000	0.000
0.25	0.000	0.000	0.360	0.560	0.480
0.5	0.000	0.000	0.460	0.720	0.380
1	0.000	0.000	0.550	0.910	0.350
3	0.003	0.007	1.133	1.507	0.757
6	0.005	0.008	1.352	1.697	0.998
12	0.006	0.009	1.283	1.518	0.884
24	0.005	0.013	0.725	1.022	0.863
48	0.005	0.010	0.376	0.562	0.466
72	0.004	0.007	0.261	0.381	0.328
Mean Rate of Solubility (µg/ml/h) ± SD	0.003 ± 0.000	0.005 ± 0.000	0.649 ± 0.014	0.888 ± 0.019	0.551 ± 0.027

Collective comparative between Piperine, GA, HAP, and FA

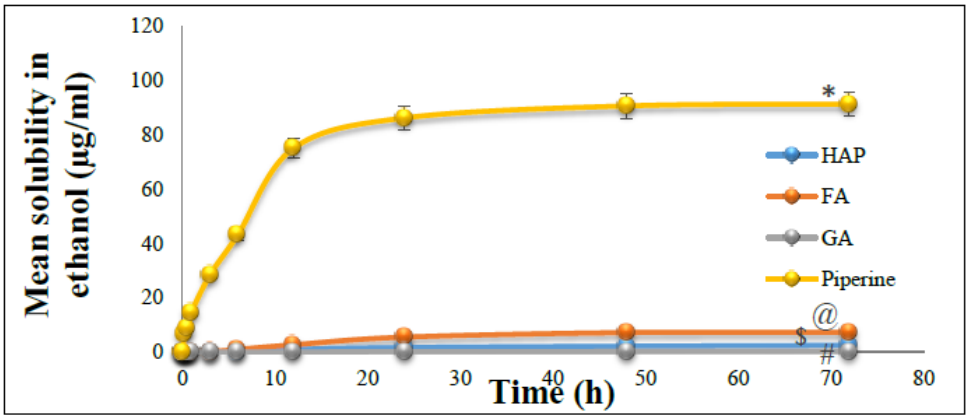

Figure S2. Comparative solubility of piperine (Pip), hydroxyapatite (HAP), folic acid (FA) and gum arabica (GA) in ethanol absolute. * shows solubility line for Pip, @ shows solubility line for FA, $ shows solubility line for HAP, and # shows solubility line for GA.

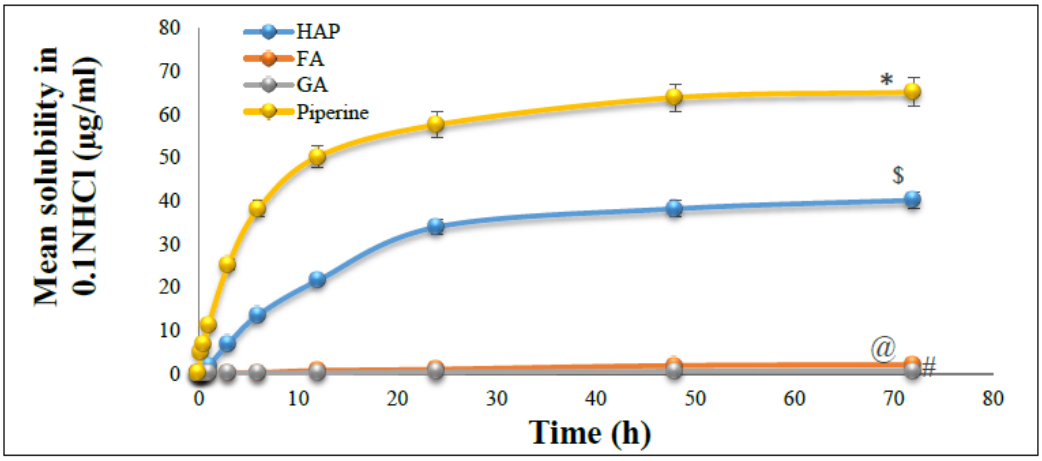

Figure S3. Comparative solubility of piperine (Pip), hydroxyapatite (HAP), folic acid (FA) and gum arabica (GA) in 0.1NHCl (pH 1.2). * shows solubility line for Pip, @ shows solubility line for FA, $ shows solubility line for HAP, and # shows solubility line for GA.

4. Release data

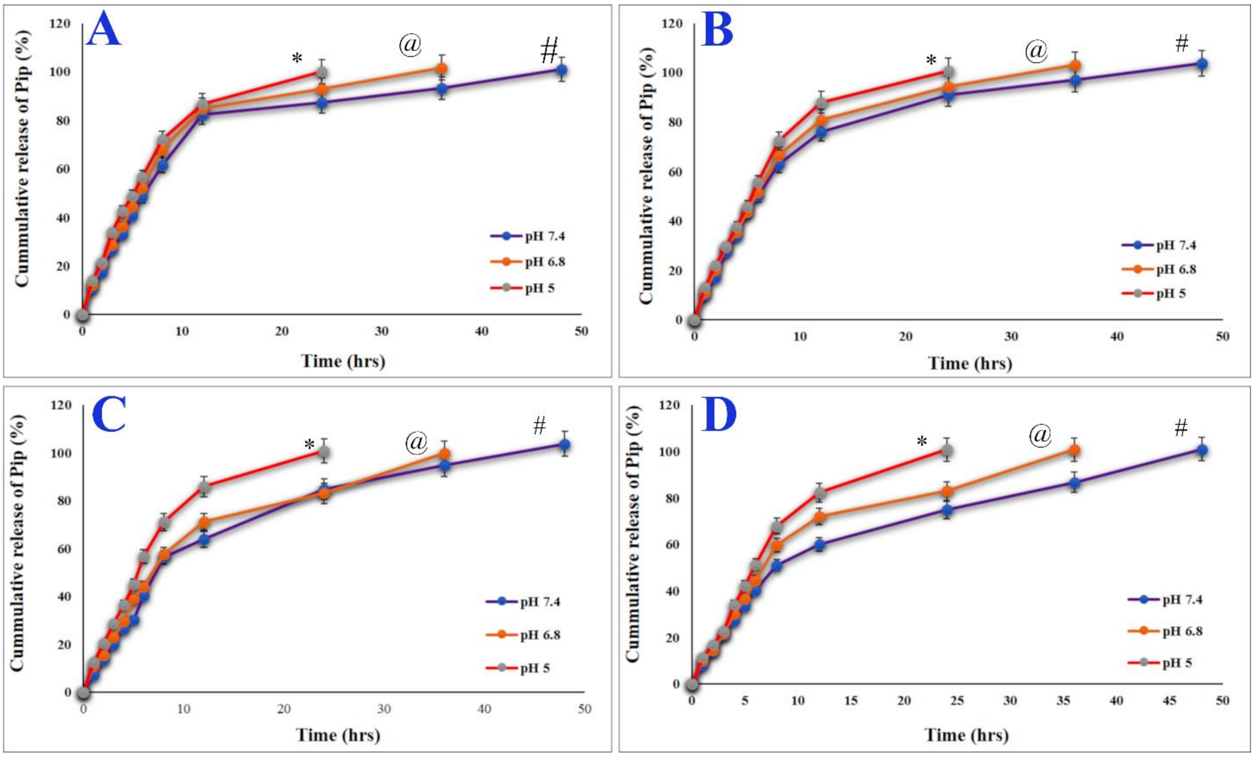

Figure S4. In vitro release Pip from PBS buffer under different pH conditions. Release profiles under pH 7.4, 6.8, and 5 for HAP-Pip7.2 (**A**), HAP-Pip9.3 (**B**), HAP-P-Pip7.2 (**C**), and HAP-P-Pip9.3 (**D**). * shows the release line at pH:5, @ shows the release line at pH:6.8, and # shows the release line at pH:7.4.

Table S9. In-vitro release criteria of Pip from various HAP-NPs structures in PBS buffer media of different pH values.

Formulation	Release Characteristics	pH of Phosphate buffer solution release medium		
		**pH 7.4**	**pH 6.8**	**pH 5**
HAP-Pip7.2	Best fitting Model		*Baker-Lonsdale*	
	RE (%)	79.45 ± 0.06	78.33 ± 0.10	73.14 ± 0.01
	MDT (h)	9.87 ± 0.01	7.80 ± 0.01	6.45 ± 0.01
	R^2	0.961	0.976	0.992

HAP-P-Pip7.2	Best fitting Model	*Baker-Lonsdale*	*Korsmeyer-Peppas*	*Baker-Lonsdale*
	RE (%)	74.89 ±0.01	70.11 ± 0.07	72.06 ± 0.09
	MDT (h)	12.06 ± 0.83	10.76 ± 0.67	6.71 ± 0.24
	R^2	0.996	0.955	0.991
HAP-Pip9.3	Best fitting Model		*Baker-Lonsdale*	
	RE (%)	80.41 ± 0.75	77.67 ± 0.80	73.01 ± 0.06
	MDT (h)	9.41 ± 0.40	8.04 ± 0.03	6.48 ± 0.06
	R^2	0.989	0.991	0.987
HAP-P-Pip9.3	Best fitting Model	*Korsmeyer-Peppas*		*Baker-Lonsdale*
	RE (%)	69.50 ± 0.06	70.29 ± 1.08	69.42 ± 0.06
	MDT (h)	14.64 ± 1.41	10.70 ± 1.00	7.34 ± 0.33
	R^2	0.948	0.947	0.986
HAP-Pip9.3-GA	Best fitting Model		*Baker-Lonsdale*	
	RE (%)	74.27 ± 0.09	76.47 ± 0.02	80.01 ± 0.04
	MDT (h)	18.53 ± 0.09	14.12 ± 0.05	9.59 ± 1.03
	R^2	0.972	0.990	0.977
HAP-P-Pip9.3-GA	Best fitting Model		*Baker-Lonsdale*	
	RE (%)	79.20 ± 1.01	79.33 ± 1.02	78.05 ± 1.09
	MDT (h)	14.98 ± 0.01	12.40 ± 0.01	10.54 ± 0.05
	R^2	0.990	0.992	0.994
HAP-Pip9.3-GA-FA	Best fitting Model		*Baker-Lonsdale*	
	RE (%)	75.62 ± 1.22	75.40 ± 1.39	77.35 ± 1.17
	MDT (h)	23.41 ± 0.60	20.67 ± 0.41	16.31 ± 0.11
	R^2	0.972	0.975	0.988
HAP-P-Pip9.3-GA-FA	Best fitting Model		*Baker-Lonsdale*	
	RE (%)	79.20 ± 1.01	79.33 ± 1.02	78.05 ± 1.09
	MDT (h)	14.98 ± 0.01	12.40 ± 0.01	10.54 ± 0.05
	R^2	0.990	0.992	0.994

5. Cytotoxicity and anticancer evaluations results for MCF7 (breast), Caco2 (colon) cancer cells lines; and WI-38 (fibroblast normal cells).

5.1. Cytotoxicity of HAP nanoparticles

Figure S5 shows that the cytotoxicity effect on cancer and normal cells was concentration, time, cell, and particle type dependent concentration (significant differences at $p < 0.5$). Increasing the concentration of HAP and HAP-P from 12.3 to 1000 μg/ml and incubation time from 48 to 72 h was significantly decreased cell viability. We see that cell viability was a little increased in normal cells compared to cancer cells (especially at high concentrations of 333 and 1000 μl). This observation means that HAP and HAP-P less toxic on normal cell compared to cancer cells. It was observed that when cells were incubated to 72 h, the cell viability was significantly decreased compared to 48 h. The minimum cell viability of 53.2% ± 0.5, 53.7% ± 0.5, and 61.2% ± 0.4 was obtained for MCF7, Caco2 cancer cells and WI-38 normal cells treated with HAP-P for 72 h, respectively. Showing that HAP-P shows more toxicity compared to HAP.

5.2. In vitro anticancer effects

Figure S6 revels the cell viability of cancers (MCF7 and Caco2) and normal cells (WI-38) was significantly ($p < 0.05$) depended on cell line, concentration, incubation time, and delivery method of Pip. Increasing incubation time from 48 h to 72 h inhibited the cell viability of all investigated cells. Increasing the concentration of all used samples from 2.4 to 200 μg/ml led to decrease cell viability, where the high reduction in viability was obtained for cells treated at 66 and 200 μg/ml, respectively. We saw the differences in the cell viability between all three cells. We found that the treatments decreased the viability of MCF7 and Caco2 compared to WI-38. Which shows less toxicity to normal cells compared to cancer cells. A strong reduction of MCF7 viability was obtained when cancer cells were treated with HAP-Pip9.3 (16.0%) and Pip (16.7%) at 200 μl and incubated for 72 h. We detected a maximum reduction of Caco2 viability when cancer cells were treated with HAP-Pip-GA-FA (21.0%) at 200 μl and incubated for 72 h. While, inhibition of WI-38 viability was observed by treating

cells with Pip (40.6%) at 200 µl and incubated for 48 and 72 h. Showing that Pip is more toxic on normal cells compared to Pip-loaded nanoparticles in different prepared nanoformulations especially those contained folic acid. This observation reflects the importance of construction of Pip delivery route as HAP-Pip-GA-FA compared to traditional application of free Pip.

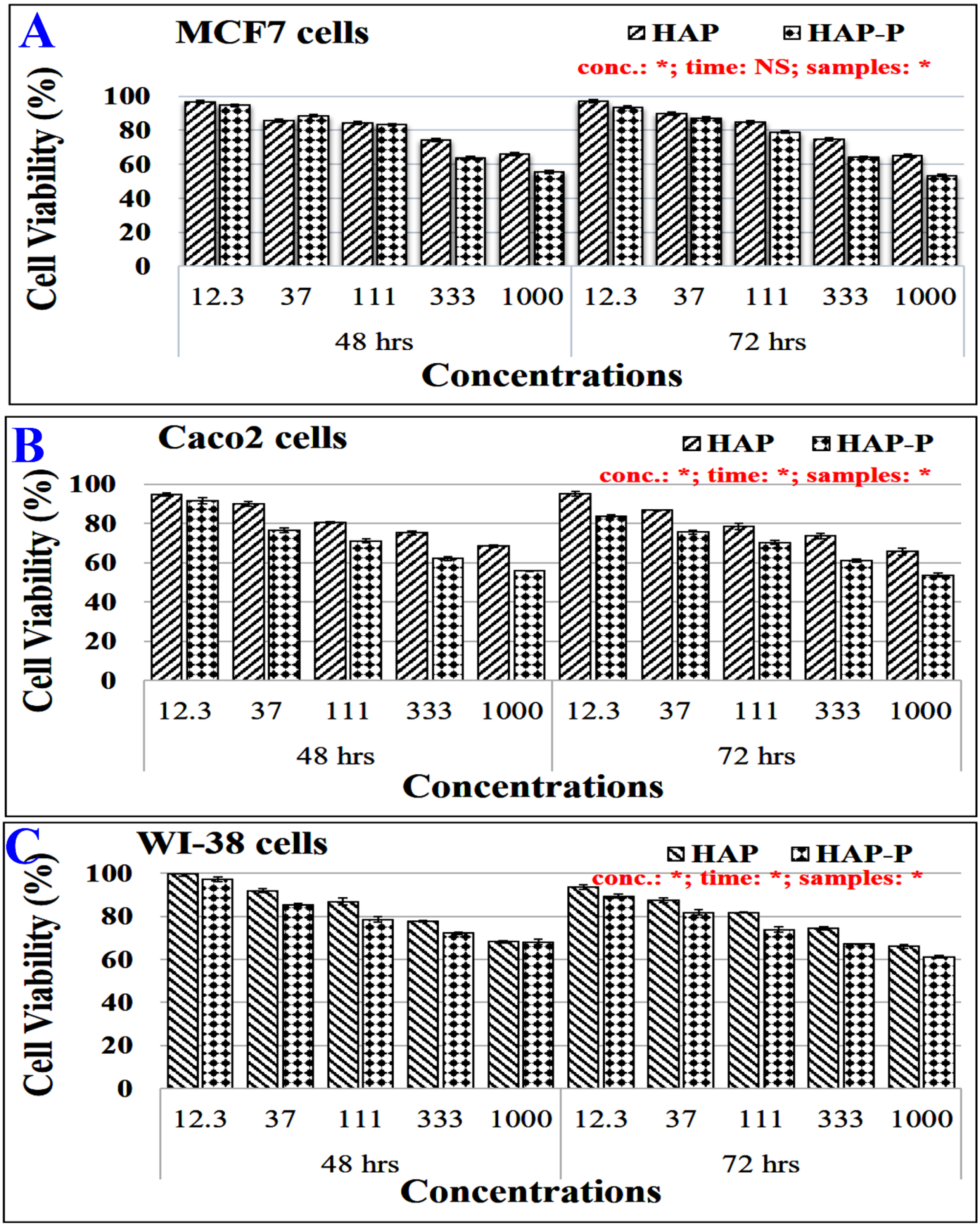

Figure S5. Cytotoxicity evaluation of HAP and HAP-P on cancer and normal cell lines after 48 and 72 h of incubation.

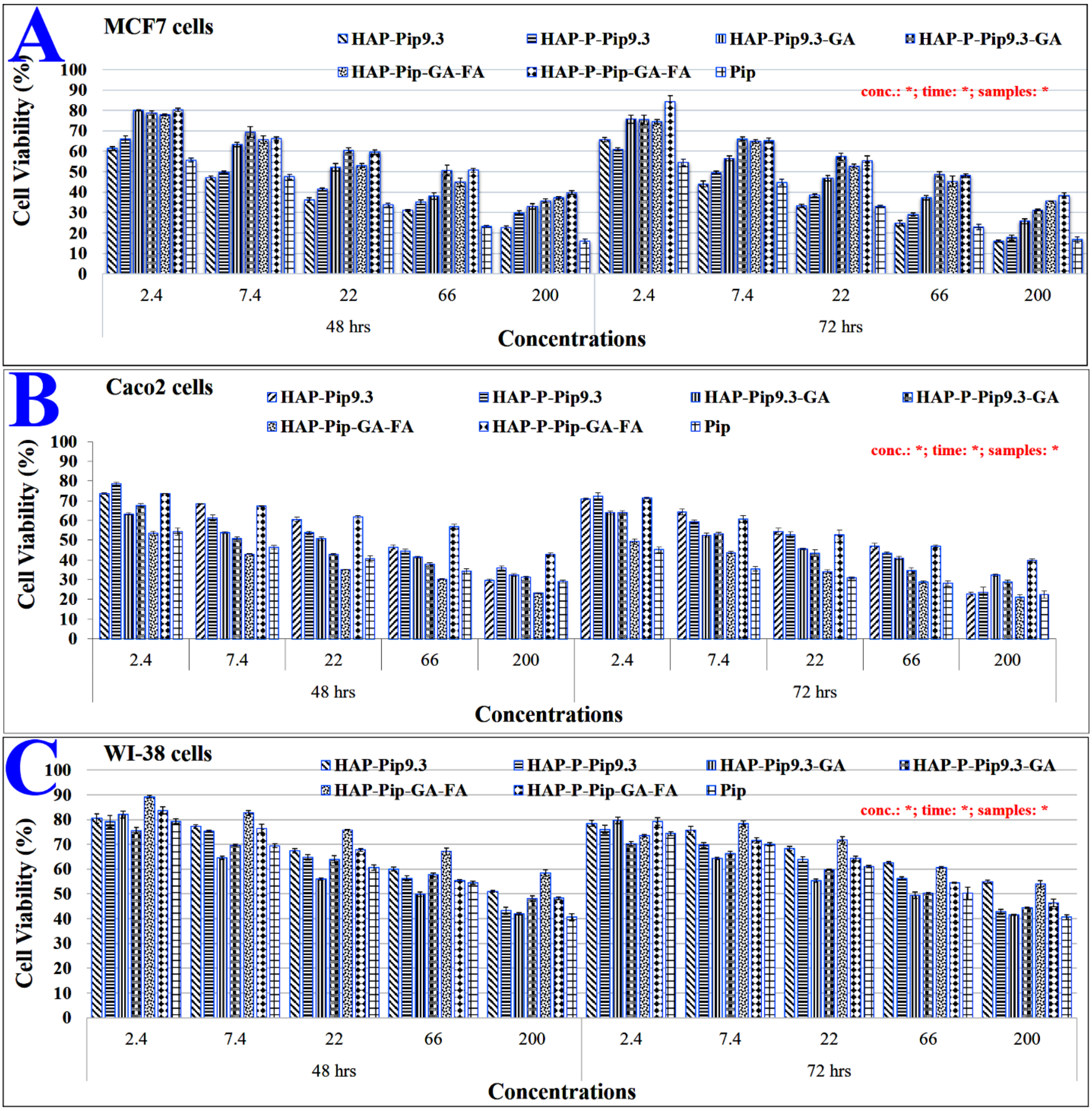

Figure S6. Cytotoxicity evaluation of all prepared materials and free Pip on cancer and normal cell lines after 48 and 72 h of incubation.

6. Anticancer observation by means of SEM

Incubation for 4 h

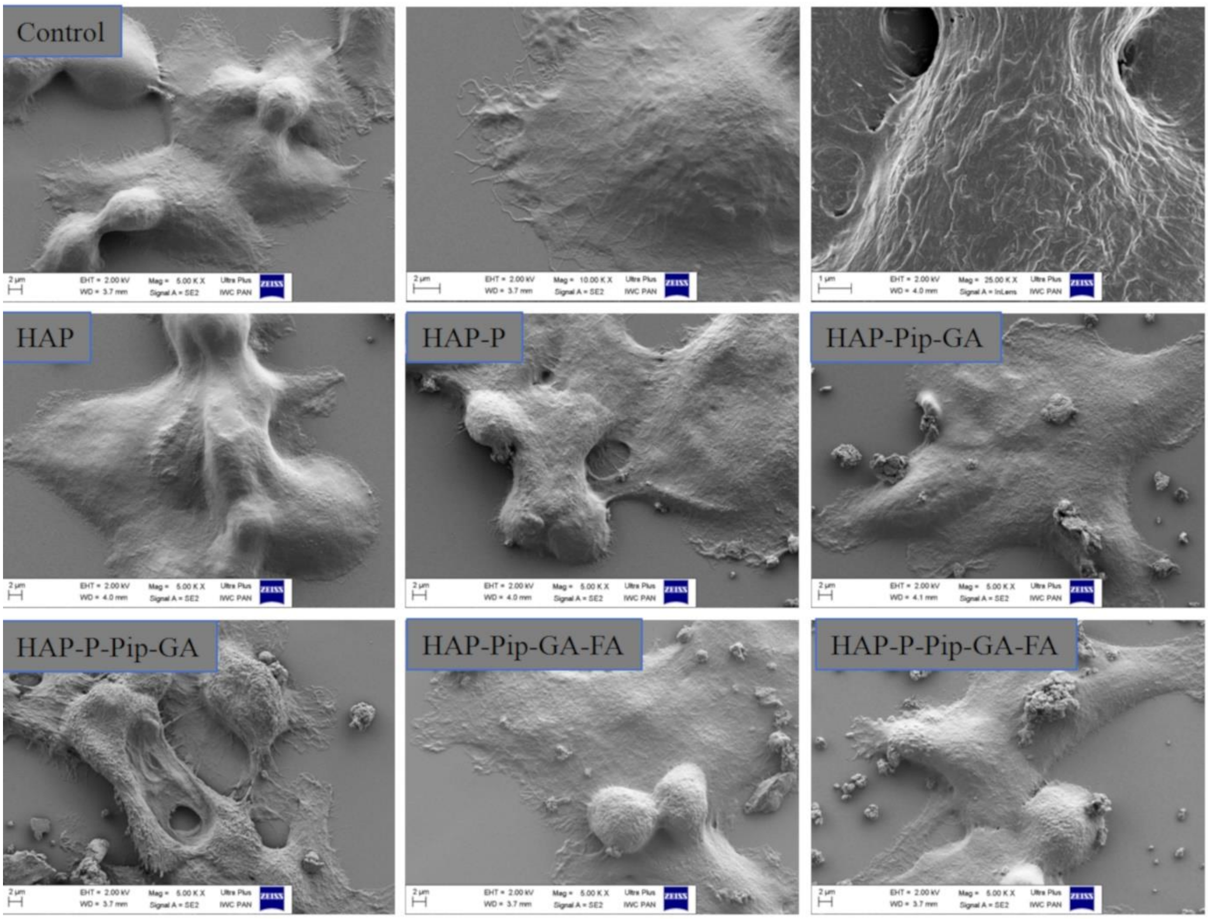

Figure S7. The anticancer effects observation by field emission scanning electron microscopy (FE-SEM) in HCT116 colon cancer cells (monolayer). The cells were treated at 200 µl of HAP and HAP-P nanoparticles and selected nanoformulations. Cells were incubated for 4 h. Note: untreated cells were used as control cells- visualized at different magnification from 5 to 25 KX- scale bar is 1 and 2 µm. Treated cells visualized with the magnification of 5KX- scale bar is 2 µm.

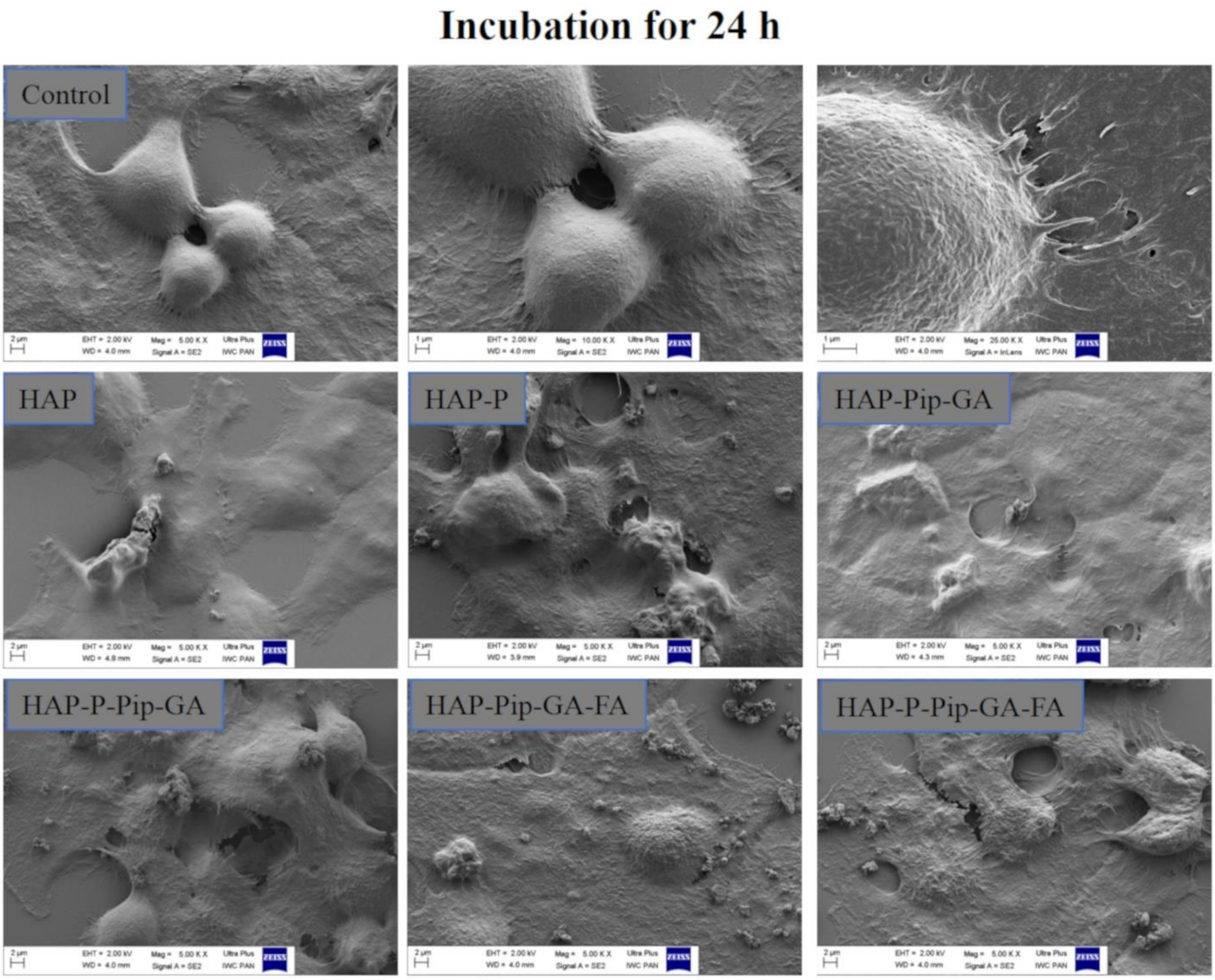

Figure S8. The anticancer effects observation by field emission scanning electron microscopy (FE-SEM) in HCT116 colon cancer cells (monolayer). The cells were treated at 200 µl of HAP and HAP-P nanoparticles and selected nanoformulations. Cells were incubated for 24 h. Note: untreated cells were used as control cells- visualized at different magnification from 5 to 25 KX- scale bar is 1 and 2 µm. Treated cells visualized with the magnification of 5KX- scale bar is 2 µm.

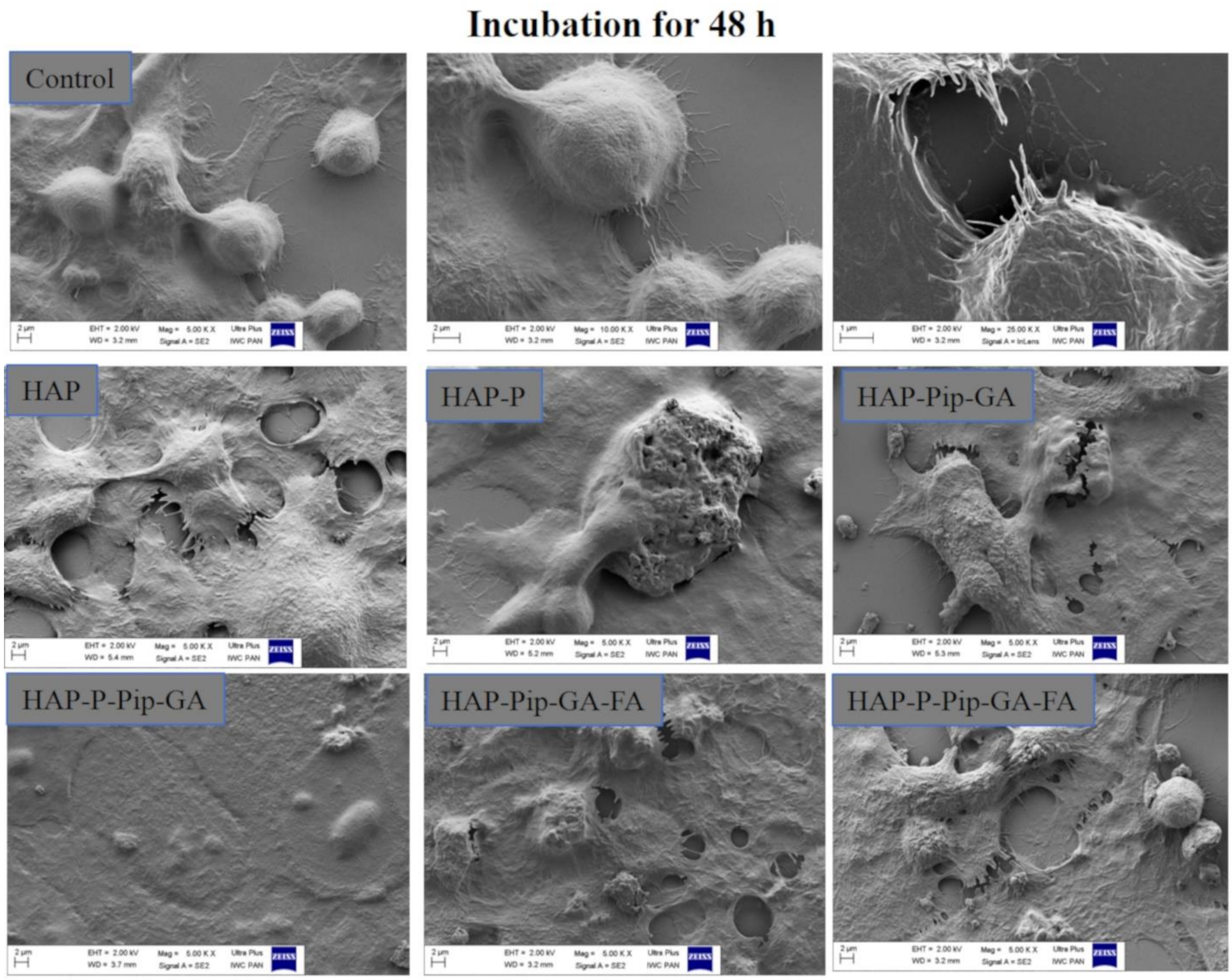

Figure S9. The anticancer effects observation by field emission scanning electron microscopy (FE-SEM) in HCT116 colon cancer cells (monolayer). The cells were treated at 200 µl of HAP and HAP-P nanoparticles and selected nanoformulations. Cells were incubated for 48 h. Note: untreated cells were used as control cells- visualized at different magnification from 5 to 25 KX-scale bar is 1 and 2 µm. Treated cells visualized with the magnification of 5KX-scale bar is 2 µm.

References

1. Baspinar, Y.; Üstündas, M.; Bayraktar, O.; Sezgin, C. Curcumin and piperine loaded zein-chitosan nanoparticles: Development and in-vitro characterisation. *Saudi Pharm. J.* **2018**, *26*, 323–334, doi:https://doi.org/10.1016/j.jsps.2018.01.010.
2. Li, K.; Liang, N.; Yang, H.; Liu, H.; Li, S. Temozolomide encapsulated and folic acid decorated chitosan nanoparticles for lung tumor targeting: improving therapeutic efficacy both in vitro and in vivo. *Oncotarget* **2017**, *8*, 111318–111332, doi:10.18632/oncotarget.22791.
3. Martins, M.T.; Paim, C.S.; Steppe, M. Development of a dissolution test for lamotrigine in tablet form using an ultraviolet method. *Braz. J. Pharm. Sci.* **2010**, *46*, 179–186.
4. Shao, B.; Cui, C.; Ji, H.; Tang, J.; Wang, Z.; Liu, H.; Qin, M.; Li, X.; Wu, L. Enhanced oral bioavailability of piperine by self-emulsifying drug delivery systems: in vitro, in vivo and in situ intestinal permeability studies. *Drug Deliv.* **2015**, *22*, 740–747, doi:10.3109/10717544.2014.898109.
5. Higuchi, T.; Connors; K.A. Phase-solubility techniques. In *Advances in Analytical Chemistry and Instrumentation*; Wiley-Interscience, New York, NY, USA, 1965, Volume 4, pp. 117–212.
6. Chen, Z.F.; Darvell, B.W.; Leung, V.W.H. Hydroxyapatite solubility in simple inorganic solutions. *Arch. Oral Biol.* **2004**, *49*, 359–367, doi:https://doi.org/10.1016/j.archoralbio.2003.12.004.
7. Wu, Z.; Li, X.; Hou, C.; Qian, Y. Solubility of Folic Acid in Water at pH Values between 0 and 7 at Temperatures (298.15, 303.15, and 313.15) K. *J. Chem. Eng. Data* **2010**, *55*, 3958–3961, doi:10.1021/je1000268.

8. Wood, E.J. *Data for biochemical research*; 3rd ed.; Dawson, R.M.C.; Elliott, D.C.; Elliott; W.H.; Jones K.M., Eds.; Oxford Science Publications, OUP, Oxford, UK 1986, pp. 580.
